# Uncertainty in the mating strategy of honeybees causes bias and unreliability in the estimates of genetic parameters

**DOI:** 10.1186/s12711-024-00898-3

**Published:** 2024-04-17

**Authors:** Tristan Kistler, Evert W. Brascamp, Benjamin Basso, Piter Bijma, Florence Phocas

**Affiliations:** 1 Université Paris-Saclay, INRAE, AgroParisTech, GABI, 78350 Jouy-en-Josas, France; 2 UMT PrADE, 84914 Avignon, France; 3 Animal Breeding and Genomics, Wageningen University & Research, P.O. Box 338, 6700 AH Wageningen, The Netherlands; 4 INRAE, UR 406 Abeilles et Environnement, 84914 Avignon, France

## Abstract

**Background:**

Breeding queens may be mated with drones that are produced by a single drone-producing queen (DPQ), or a group of sister-DPQs, but often only the dam of the DPQ(s) is reported in the pedigree. Furthermore, datasets may include colony phenotypes from DPQs that were open-mated at different locations, and thus to a heterogeneous drone population.

**Methods:**

Simulation was used to investigate the impact of the mating strategy and its modelling on the estimates of genetic parameters and genetic trends when the DPQs are treated in different ways in the statistical evaluation model. We quantified the bias and standard error of the estimates when breeding queens were mated to one DPQ or a group of DPQs, assuming that this information was known or not. We also investigated four alternative strategies to accommodate the phenotypes of open-mated DPQs in the genetic evaluation: excluding their phenotypes, adding a dummy pseudo-sire in the pedigree, or adding a non-genetic (fixed or random) effect to the statistical evaluation model to account for the origin of the mates.

**Results:**

The most precise estimates of genetic parameters and genetic trends were obtained when breeding queens were mated with drones of single DPQs that are correctly assigned in the pedigree. However, when they were mated with drones from one or a group of DPQs, and this information was not known, erroneous assumptions led to considerable bias in these estimates.

Furthermore, genetic variances were considerably overestimated when phenotypes of colonies from open-mated DPQs were adjusted for their mates by adding a dummy pseudo-sire in the pedigree for each subpopulation of open-mating drones. On the contrary, correcting for the heterogeneous drone population by adding a non-genetic effect in the evaluation model produced unbiased estimates.

**Conclusions:**

Knowing only the dam of the DPQ(s) used in each mating may lead to erroneous assumptions on how DPQs were used and severely bias the estimates of genetic parameters and trends. Thus, we recommend keeping track of DPQs in the pedigree, and not only of the dams of DPQ(s). Records from DPQ colonies with queens open-mated to a heterogeneous drone population can be integrated by adding non-genetic effects to the statistical evaluation model.

**Supplementary Information:**

The online version contains supplementary material available at 10.1186/s12711-024-00898-3.

## Background

Although mating control is essential to the genetic improvement of a honeybee breeding program [1], its practical application is not straightforward, due to the behavioral and reproductive specificities of queens. Indeed, natural mating occurs during flight, typically at a height of a few tens of meters, where drones and young queens from several kilometers around gather together [2]. In order to control the genetic origin of mates in a breeding program, virgin queens and mature drones can be isolated geographically on mating stations, or artificial insemination can be used.

At the mating stations used in selective breeding, a group of sister drone-producing queens (DPQs) descending from a single dam is usually used to produce all the drones at the mating station. This group is referred to as a pseudo-sire (PS) and is registered in the pedigree. To ensure high mating success, the group usually comprises between four and 12 DPQs (personal communication from the French Royal Jelly Producers’ Association (GPGR) and island mating in the Beebreed dataset [3]), depending on the number of virgin queens to be mated. This is referred to as PS mating.

The alternative, artificial insemination, enables greater mating control. In particular, drones used to mate a virgin queen can be taken from a PS composed of very few sister-DPQs or even from a single DPQ (for example in [4]). In the latter case, this is referred to as single sire (SS) mating. Compared to PS mating, SS mating results in higher relatedness among the workers and offspring queens in the colony. When correctly accounted for in the pedigree, SS mating should result in estimates of genetic parameters and breeding values with lower standard errors than PS mating, for which the precise origin of drones cannot be distinguished among the sister-DPQs and needs to be derived probabilistically [5, 6]. However, when artificial insemination is used, honeybee breeders often record only the dam of the DPQ(s) and provide no information on the number of sister-DPQs, even when only one DPQ is involved, and in this latter case, they also do not record the identity of the DPQ used.

Furthermore, it is common that selective breeding programs also include open mating, where virgin queens are allowed to mate freely with drones from the surrounding area (for example, in Italy [7]). In particular, DPQs are often open-mated because this reduces management costs (for example, in France [8]). If the contribution of DPQs to the breeding population is limited to producing drones, then their mates do not affect the genetic evaluation because drones are haploid individuals born from unfertilized eggs and thus do not carry genes from their dam’s mate. However, when artificial insemination is used, these DPQs are often phenotyped and used for drone production after phenotypic selection. In that case, the mates of DPQs affect genetic evaluation as they are the sires of the workers of DPQ colonies whose records are used for genetic evaluation.

For most traits, the phenotypes of a colony are expected to be affected by both the queen and her worker group, as revealed by genetic parameter estimates obtained with real data [9–12]. Alongside environmental effects, colony performance should therefore be partitioned into two genetic effects: a worker genetic effect expressed by the worker group, and a queen genetic effect expressed by the queen [13, 14]. Therefore, rather than a single genetic variance, three genetic parameters need to be estimated: the variances of queen effects, of worker effects, and their covariance. A reliable estimation of genetic parameters requires data and pedigree records from a large population of genetically well-connected apiaries. Unfortunately, most honeybee breeding programs use small selection nuclei of ten to a few tens of breeding queens [8, 11, 15, 16]. In addition, queens only mate before they lay their first egg, and will normally never mate afterwards. Thus, the reproduction mode of honeybees condemns a queen to being evaluated with her single mate, regardless of whether it is a PS or SS. This adds to the difficulty of disentangling the genetic contribution of workers to the colony phenotype from that of the queen. This is different from other livestock species, where females can have offspring from several known mates, but even in that case, disentangling direct effects from maternal effects remains difficult [17, 18].

Du et al. [19] recently explored the effects of data structure on the estimates of genetic parameters in simulated unselected honeybee populations. Among other parameters, they varied the proportion of missing phenotypes, as well as the ratio of controlled and uncontrolled mated queens, with drones always originating from the closed selection nucleus. They demonstrated the importance of the proportion of controlled mating for the accuracy of the estimates of genetic parameters and obtained unbiased estimates if at least 20% of the colony records were used. However, they explored neither the impact of SS vs PS mating, and the consequences of erroneous assumptions when the true mating strategy is unknown, nor how to model the effect of the open mating of DPQs with colony phenotypes.

With the increasing number of honeybee breeding plans worldwide, colony records on queens with diverse mating strategies often need to be considered in genetic analyses. Here, we used simulation to investigate the impact of the mating strategy of breeding queens and open-mated potential DPQs, and of sire modeling, on the bias and standard error of the estimates of genetic parameters and genetic trends.

## Methods

For all the scenarios, we simulated colonies with different mating strategies for inseminated breeding queens (BQs) and for open-mated DPQs. BQs were mated to drones that were produced by either a single DPQ or a group of three sister-DPQs, while DPQs were mated to either a homogeneous or a heterogeneous open-mating drone population. We inspected the bias and standard errors of the estimates of genetic parameters and genetic trends, using either correct or incorrect assumptions for the mating strategy.

In simulation Set I, we focused on the controlled mating of BQs, assuming that it is either known or unknown whether drones used in the mating originate from a single DPQ or from a group of three sister-DPQs. In simulation Set II, we focus on the open-mating of DPQs, using four alternative strategies to account for the heterogeneity of the open-mating drone population in the statistical analysis. Table 1 gives an overview of the scenarios used in the two simulation sets and the scenarios used to estimate the genetic parameters and genetic trends.

**Table 1:** Simulation and estimation scenarios

Simulation scenarios			Estimation scenarios	
Set	Mating strategy		Sire modeling	
	Controlled mating of BQs	Open mating of DPQs	Controlled mating of BQs	Open mating of DPQs
I	SS	Homogeneous open mating drone population	C_SS_P_	O_PS_P_
			C_dummySS_P_/DPQdam	
			C_dummySS_P_/Q	
			C_PS_P_	
	PS		C_SS_P_	O_PS_P_
			C_dummySS_P_/DPQdam	
			C_dummySS_P_/Q	
			C_PS_P_	
II	SS	Heterogeneous open mating drone population	C_SS_P_	O_NoPheno
				O_TwoPS_P_
				O_FixedGroup
				O_RandGroup

Controlled mating strategy in the simulation: SS (single sire) and PS (pseudo-sire) mating

Sire pedigree modeling for controlled mating: C_SS_P_, C_dummySS_P_/DPQdam, C_dummySS_P_/Q, C_PS_P_: in the pedigree, controlled mated queens are assigned, respectively, single sires, dummy single sires per dam of DPQ(s), dummy single sires per mated queen, pseudo-sires

Sire modeling for open mating: O_NoPheno: open-mated DPQs’ colony phenotypes were excluded from the genetic analysis; O_PS_P,_ O_TwoPS_P_: in the pedigree, open-mated DPQs are assigned, respectively, a single open-mating pseudo-sire, or one for each open-mating drone subpopulation (initial BQs and each half of the DPQ); O_FixedGroup or O_RandGroup: the effect of the drone subpopulation mating DPQs was accounted for by adding a fixed or a random non-genetic effect in the statistical model describing the phenotypes

### Simulation: founder population, mating, selection, and reproduction

**Fig. 1:**
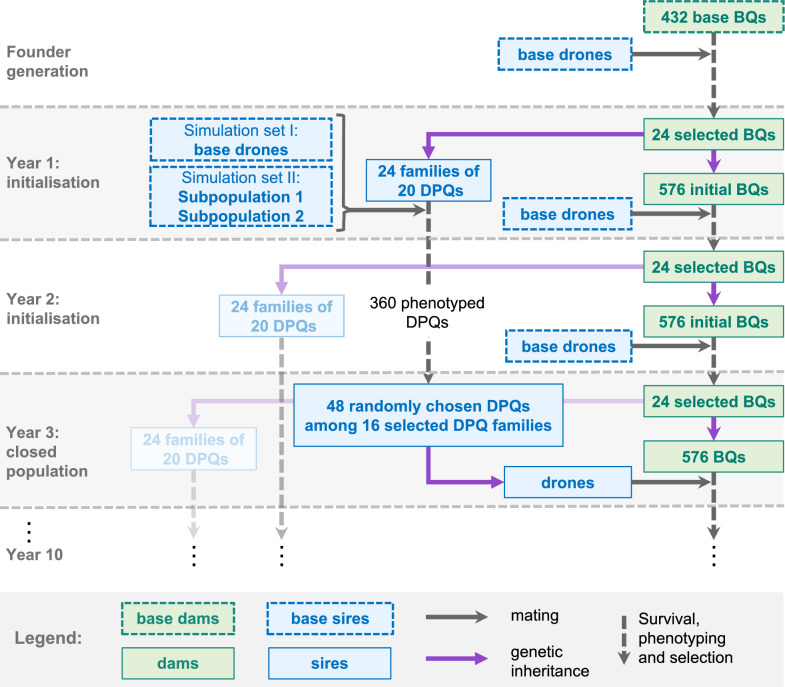
Simulation process. *BQ* breeding queen, *DPQ* drone-producing queen. BQ refers to queens that were candidates for selection, and “selected BQ” to the selected dams. Because of overlapping generations, the selection of DPQ families is completely shown only for the first event (years 1 to 3). See main text for a description of the simulation process

Figure 1 summarizes the simulation of all generations of queens, drones, and worker groups. The infinitesimal model used followed the method proposed by Kistler et al. [20] and the main equations are shown in Additional file 1: Text S1.

The simulation started with the generation of founders, followed by 2 initialization years in which BQs were open mated, since a first generation of DPQs would be available for drone production only in year 3 (Fig. 1). The initial founder generation consisted of 432 unrelated, non-inbred, and open-mated base BQs. The term “BQ” is used to indicate queens that were candidates for selection, and “selected BQ” to refer to the selected dams. Base BQs were mated with base drones to produce worker groups to form colonies. Phenotypes were obtained from the 432 colonies, followed by selection of the 24 BQs with the highest colony phenotypes to produce new offspring queens.

From year 1 and in each subsequent year, each of the 24 selected BQs produced 24 offspring BQs and 20 DPQs. Virgin DPQs were open-mated, as were all following generations of DPQs. In year 2 and each subsequent year, a mortality event was simulated by randomly eliminating 25% of all colonies. Phenotypes were obtained from the remaining 75% of BQ and DPQ colonies, which correspond, respectively, to 18 and 15 sister queens phenotyped per dam. After phenotyping, each year, one BQ from each sister-group was selected based on its colony phenotype to produce new offspring queens. Thus, in the dam path, selection was within the maternal family with one replacement queen per maternal sister-group. In the sire path, two-thirds of the dams of DPQs were selected each year based on the mean phenotype of their DPQ offspring (across-family selection). After a second winter mortality event, three DPQs were randomly chosen per selected sister-group in year 3 and each subsequent year. In year 3 and each subsequent year, BQs were mated with drones produced by these chosen DPQs, which closed the breeding population. The same selection and mating procedure were repeated until year 10. The resulting generation interval was 1.5 years (1 year on the dam path and 2 years on the sire path).

Other simulation parameters included the number of drones that mate each queen, which was fixed at 8. These eight drones contributed equally to the genetic effect of the worker group in a colony.

In our base genetic parameter set, founder BQs had breeding values that were drawn from a bivariate normal distribution centered on zero, with a genetic variance for queen effects (${\upsigma }_{{\text{Q}}}^{2}$) and worker effects (${\upsigma }_{{\text{W}}}^{2}$) both equal to one-third of the residual variance (${\upsigma }_{{\text{e}}}^{2}$), and a null genetic correlation. Additional simulations were run using three other genetic parameter sets (see Additional file 2: Table S1). The variance of worker effects was doubled in genetic parameter sets 2 and 4. The genetic correlation between worker and queen effects (${{\text{r}}}_{{\text{WQ}}}$) was zero in the two first genetic parameter sets and − 0.5 in the last two. This range of parameters represents typical estimates for honeybee production and behavioral traits [9–12]. The breeding values of base drones were also drawn from a normal distribution, but with a halved (co)variance matrix to simulate haploidy. In simulation Set I, their breeding values were centered on **0**, while in simulation Set II, one half of the drones mating with DPQs had their BV centered on $\mathbf{-}{\varvec{\upalpha}}$ and the other half on $\mathbf{+}{\varvec{\upalpha}}$, as described below.

### Simulation Set I: sire modeling for the controlled mating of breeding queens

In simulation Set I, we explored the impact of the sire modeling for controlled mating on the genetic analysis. Two controlled mating strategies for BQs were considered in the simulation. The first strategy was SS mating, where a single DPQ was chosen at random from three sister-DPQs to produce all the drones that mate a single BQ. The second strategy was PS mating, where the three sister-DPQs formed a PS. The PS drone pool was obtained for each mating by drawing at random (with replacements) a DPQ eight times from the three sister-DPQs to produce the eight drones used for mating. In both strategies, the contribution of each group of sister-DPQs to the total drone pool that mated with all the BQs was balanced. All DPQ generations were open-mated with a homogeneous (or unstructured) drone population descending from the same base population as the BQs. Thus, their BVs were drawn from a single bivariate normal distribution centered on **0**.

**Fig. 2:**
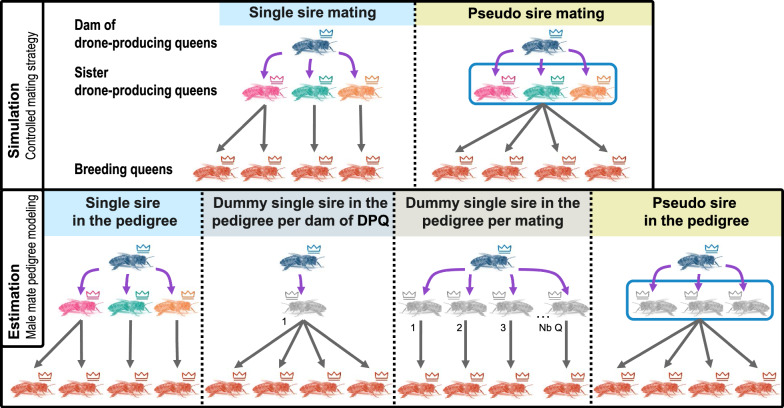
Sire modeling scenarios for controlled mating. In simulation Set I, either single sire mating (all drones mating with a queen are produced by a single drone-producing queen, DPQ) or pseudo sire mating (all drones mating with a queen are produced by a group of three sister-DPQs) was used. The dam of DPQ(s) was always correctly assigned. Regardless of the true (simulated) mating strategy adopted, four hypothetical scenarios were used to derive the sire pedigree, and to simulate different ways of handling uncertainty in the true mating strategy. First, in the ‘single sire in the pedigree’ modeling, individual DPQs were assigned exactly as they were used for mating when single sire mating was the controlled mating strategy in the simulation; alternatively, one of the three sister DPQs making up the pseudo sire was randomly assigned in the pedigree when pseudo sire mating was used for the simulation. Second, under the ‘dummy single sire in the pedigree per dam of DPQ’ scenario, a unique dummy DPQ was assigned to all queens that mated with drones from the same dam of DPQs. Third, under the ‘dummy single sire in the pedigree per mating’ scenario, one dummy DPQ was assigned to the pedigree for each mating. Lastly, in the ‘pseudo sire in the pedigree’ model, a pseudo sire made of three sister DPQs was assigned to the pedigree

In the estimation, the dam of the DPQ(s) that mated with BQs was always correctly assigned, but four different sire-modeling scenarios were considered to study the consequences of not knowing whether one or more DPQs were used (Table 1 and Fig. 2). These four sire-modeling scenarios were used for each of the true mating strategies (SS and PS). First, under the C_SS_P_ scenario (where “C” refers to “controlled mating”, and the subscript to how the sire was included in the pedigree), individual DPQs were assigned in the pedigree exactly as they were used for mating when SS was the controlled mating strategy used for the simulation; alternatively, one of the three sister-DPQs making up the PS was assigned randomly in the pedigree when PS mating was used for the simulation (PS). Second, under the “C_dummySS_P_/DPQdam” scenario, a single DPQ was assigned to all queens mated with drones from the same dam of DPQs. Third, under the “C_dummySS_P_/Q” scenario, a different dummy DPQ was assigned in the pedigree to each controlled-mated queen. Lastly, under the C_PS_P_ scenario, a dummy sire was assigned in the pedigree and corresponded to a PS made up of three sister-DPQs, as described by Brascamp and Bijma [6].

For all four scenarios, the contribution of open-mating drones to the colony phenotypes of base and initial BQs and all DPQs was modeled by genetic effects through the pedigree, as follows: a single group of 100 unknown non-inbred and unrelated DPQs (an open-mating PS_P_) was assumed to produce all open-mating drones and was uniquely assigned in the pedigree files. The open-mating PS_P_ was modeled as a group of individuals, and thus its coefficients in the relationship matrix were divided by the number of DPQs that it was assumed to comprise (see Additional file 3: Text S2, for more details).

### Simulation Set II: sire modeling for the open mating of drone-producing queens

In simulation Set II, we explored the impact of the sire modeling of worker groups from open-mated queens on the genetic analysis. In this simulation set, we only considered SS mating for BQs (except base and initial BQs at the start of the simulation). However, DPQs were mated with a heterogeneous (or structured) open-mating drone population (unlike the homogeneous population in simulation Set I). This heterogeneous drone population consisted of two subpopulations, with a mean genetic level that differed by approximately one genetic standard deviation of queen effects. DPQ sister-groups were randomly allocated to mate with drones from one of the two subpopulations. This could, for example, reflect a breeding population in which DPQs are mated in two different geographic areas, each with a different drone population.

In the estimation, we assumed SS_P_ for BQs (considering that it is known that SS mating was indeed used), except for base and initial BQs. For DPQs, we considered four scenarios to model the effect of the drone subpopulations mating with them. First, under the O_NoPheno scenario (where “O” refers to “open mating”), we eliminated colony phenotypes of DPQs from the performance file. Deleting these records removed the impact of drone subpopulations from the data, except for a weak DPQ family selection that had occurred. It served as a baseline scenario for the comparison to the other scenarios that integrate these additional phenotypes. Second, in the O_TwoPS_P_ scenario, each half of the DPQs was assigned to a distinct open-mating PS_P_ in the pedigree to account for the drone subpopulation with which they were mated. In the last two scenarios, we accounted for the effect of the drone subpopulations by adding either a fixed (O_FixedGroup) or a random (O_RandGroup) non-genetic effect in the statistical model describing the phenotypes. For base and initial BQs in all four scenarios, and also for the DPQs in the last two scenarios, we assigned a unique open-mating PS_P_ as mate (as in simulation Set I, see Additional file 3: Text S2).

Two hundred replicates were run for all Set I and Set II simulation scenarios.

### Genetic evaluation using a mixed model with queen and worker effects

Pedigree and colony records were used to obtain a single retrospective estimation of the genetic parameters and breeding values. The vector of phenotypes, $\mathbf{y}$, was described using a linear mixed model with worker and queen effects [13, 14]:

$$\mathbf{y}=\mathbf{X}\mathbf{b}+{\mathbf{Z}}_{\mathbf{w}}{\mathbf{a}}_{\mathbf{w}}+{\mathbf{Z}}_{\mathbf{q}}{\mathbf{a}}_{\mathbf{q}}+\mathbf{e},$$

where $\mathbf{b}$ is the vector of fixed year effects with the corresponding incidence matrix $\mathbf{X}$, ${\mathbf{a}}_{\mathbf{w}}$ is the vector of worker effects with the incidence matrix ${\mathbf{Z}}_{\mathbf{w}}$, ${\mathbf{a}}_{\mathbf{q}}$ is the vector of queen effects with the incidence matrix ${\mathbf{Z}}_{\mathbf{q}}$, and $\mathbf{e}$ is the vector of residuals.

For the simulation Set II in the scenarios O_FixedGroup and O_RandGroup, an additional fixed or random effect was added to the estimation model. This effect had three levels: one for the base drones mating with base and initial BQs (for the first 3 years of the breeding scheme), and one for each of the two drone subpopulations mating with DPQs.

The performance file in each analysis included all 8352 records on BQ and DPQ colonies. All queens had a line in the input pedigree file, providing the identity of their dam (unknown for base and initial BQs) and their mate, which was either a single DPQ or a dummy individual, i.e. a PS. Additional columns described the mate by identifying the DPQ(s)’s dam, the number of DPQs it comprised, as well as the number of drones it contributed to the mating (eight). The pedigree of the mate was either known (for controlled mating) or unknown (for open mating). Thus, the complete pedigree file contained entries for queens, sires, and worker groups. The pedigree was then used to compute the matrix of additive genetic relationships and its inverse, according to Brascamp and Bijma [6, 21]. The software developed by Brascamp to generate the complete pedigree file and the inverse relationship matrix was made publicly available (10.5281/zenodo.7951334).

We used AIREMLF90 from the BLUPF90 package [22] to estimate the genetic parameters and solve the best linear unbiased prediction (BLUP) equations using our performance file and honeybee-specific inverted relationship matrix. The starting values used are shown in Additional file 2: Table S2.

### Summary statistics for the estimates of genetic parameters and breeding values

The bias on genetic variances was expressed relative to the true values and given in percentages. However, the bias on the estimated ${{\text{r}}}_{{\text{WQ}}}$ was not divided by the true genetic correlation, which was zero, and therefore it was expressed in absolute values. The true genetic trend was calculated as the regression coefficient of average true breeding values (BVs) of BQs over years, from the fifth generation of the breeding program (after the selection nucleus became closed) to the last tenth generation of selection. The estimated genetic trend was derived similarly but based on estimated breeding values (EBVs) rather than BVs. Over or underestimates of the genetic trend were calculated as the difference between the estimated and true trend, relative to the true trend.

All statistical analyses were run under R [23], using several packages for data formatting [24, 25], and production of the figures [24, 26, 27].

## Results

First, we describe the results of the genetic analyses using various scenarios for the sire modeling of controlled mated BQs (simulation Set I). We explore the relevance of knowing whether the three sister-DPQs of each known dam of DPQs had been used as a PS or SS, and for SS, which DPQ in particular had been used in each mating. Second, we show the results of the genetic analyses for the sire modeling of open-mating DPQs with colony phenotypes, when they were mated with a heterogeneous drone population (simulation Set II). In this second part, we compare the results obtained either (i) by ignoring the colony phenotypes of DPQs, or (ii) by accounting for the effect of drone subpopulations in the pedigree, or (iii) by adding an additional non-genetic effect in the evaluation model describing the phenotypes.

### Results across all scenarios

Across all scenarios, no strong biases were observed when the male mate pedigree of BQs was known and correctly modeled (SS_P_ for SS mating and PS_P_ for PS mating), and when the effect of drone subpopulations in open mating was accounted for by an additional non-genetic effect in the statistical model. However, in the other pedigree modeling scenarios, the bias or standard errors (or both) that affect the genetic (co)variance components could be considerable, while they were almost always small on the estimated variance of residual effects (${\widehat{\upsigma }}_{{\text{e}}}^{2}$). Some of these marked biases resulted from under or overestimated variances being compensated for by over or underestimated covariances. In addition, within a replicate, there was a tendency to both under- (or over-) estimate ${\upsigma }_{{\text{W}}}^{2}$ and ${\upsigma }_{{\text{Q}}}^{2}$.

The results focus on the base genetic parameter set in which ${\upsigma }_{{\text{W}}}^{2}$ = ${\upsigma }_{{\text{Q}}}^{2}$ and ${{\text{r}}}_{{\text{WQ}}}$ = 0. The results obtained for the other genetic parameter sets are presented in Additional file 2: Tables S1 to S8. Within a scenario, the errors on the estimated genetic parameters or genetic trends were similar across all genetic parameter sets (except for expected differences, e.g., doubling ${\upsigma }_{{\text{W}}}^{2}$ reduced the relative errors on its estimate (${\widehat{\upsigma }}_{{\text{W}}}^{2}$). The main differences between genetic parameter sets were that errors on the estimated genetic correlation between worker and queen effects (${\widehat{{\text{r}}}}_{{\text{WQ}}}$) were smaller for ${{\text{r}}}_{{\text{WQ}}}$ = − 0.5 than for ${{\text{r}}}_{{\text{WQ}}}$ = 0, and the tendency towards both the under-(and over-) estimation of genetic variances was stronger for ${{\text{r}}}_{{\text{WQ}}}$ = − 0.5. For all scenarios, at least 98% of the 200 replicates converged, except for simulation Set II with an equal variance for worker and queen effects, ${{\text{r}}}_{{\text{WQ}}}$ = − 0.5, and a random non-genetic effect for the open-mating drone subpopulation, where 24% of the replicates failed to converge.

### Simulation Set I: sire modeling for the controlled mating of breeding queens

#### Simulating SS or PS controlled mating and estimating genetic parameters accordingly

**Fig. 3:**
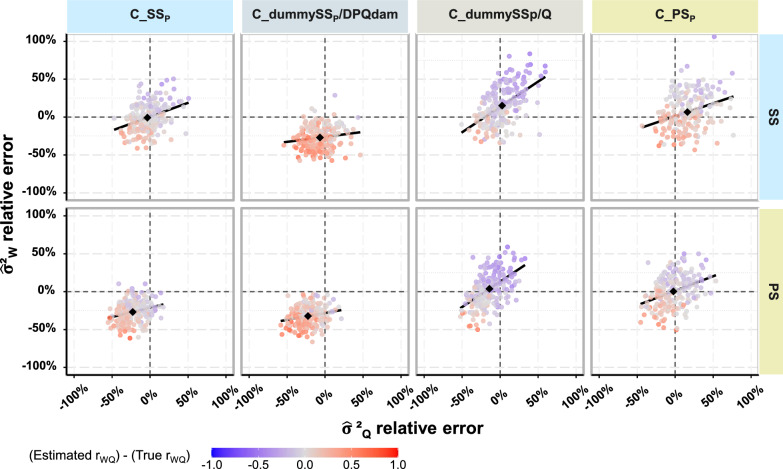
Errors on genetic parameter estimates for sire modeling scenarios for controlled mating and parameter set 1. Black diamond-shaped points indicate the relative bias on genetic variances, and black lines the regression lines of relative errors of variance estimates of worker effects on that of queen effects. The first row of graphs is from scenarios using single sire mating (SS) as the controlled mating strategy in the simulation, while the second row is from scenarios using, pseudo sire mating (PS). From left to right, results were obtained with sire pedigree modeling scenarios for controlled mating assigning: single sires (C_SS_P_); a dummy single sire per dam of DPQs (C_dummySSp/DPQdam); a dummy single sire for each mating (C_dummySS_P_/Q); and pseudo sires (C_PS_P_)

When the mating of BQs was correctly modeled in the pedigree, no strong biases on (co)variance estimates were observed. However, across scenarios, we still observed a trend towards a small underestimation of the variance of queen effects (− 3%, Table 2) and a more marked underestimation for the genetic trend (Fig. 3) on queen effects (− 6% of the true genetic trend with ${\upsigma }_{{\text{W}}}^{2}$ = 10 and ${{\text{r}}}_{{\text{WQ}}}$ = 0, see Additional file 2: Table S4).

**Table 2:** Errors in the genetic parameter estimates when male mates for controlled mating were correctly modeled in the pedigree

Simulation	Errors in the genetic parameters estimates							
Controlled mating strategy	${\widehat{\upsigma }}_{{\text{W}}}^{2}$			${\widehat{\upsigma }}_{{\text{Q}}}^{2}$			${\widehat{{\text{r}}}}_{{\text{WQ}}}$	
	Relative bias (%)	Relative SE (%)	% strong deviations	Relative bias (%)	Relative SE (%)	% strong deviations	Bias	SE
SS	− 0.81	18.46	27	− 3.45	17.50	27	0.011	0.152
PS	0.20	20.79	37	− 2.21	19.55	30	0.013	0.165

Controlled mating strategy: SS (single sire) and PS (pseudo-sire) mating

${\widehat{\upsigma }}_{{\text{W}}}^{2}$, ${\widehat{\upsigma }}_{{\text{Q}}}^{2}$ and ${\widehat{{\text{r}}}}_{{\text{WQ}}}$: estimates of the genetic variances and correlation for worker and queen effect

Strong deviations are those that differ by more than 20% from the true values. The relative bias was calculated as $\left(\frac{1}{{n}_{rep}}\sum_{1}^{{n}_{rep}}\left(\frac{{\widehat{\upsigma }}_{{\text{A}}}^{2}-{\upsigma }_{{\text{A}}}^{2}}{{\upsigma }_{{\text{A}}}^{2}}\right)\cdot 100\right)$. The relative standard error was the standard deviation across replicates of the relative difference between estimates and the true value

About 30% of the genetic variance estimates deviated by more than 20% from their true values (Table 2). In this genetic parameter set with ${\upsigma }_{{\text{W}}}^{2}$ = ${\upsigma }_{{\text{Q}}}^{2}$ and ${{\text{r}}}_{{\text{WQ}}}$ = 0, the relative standard error of ${\widehat{\upsigma }}_{{\text{W}}}^{2}$, averaged across both mating strategies, was almost equal (20%) to that of ${\widehat{\upsigma }}_{{\text{Q}}}^{2}$ (19%). However, with ${\upsigma }_{{\text{W}}}^{2}$ = ${\upsigma }_{{\text{Q}}}^{2}$, but ${{\text{r}}}_{{\text{WQ}}}$ = − 0.5, the relative standard error of ${\widehat{\upsigma }}_{{\text{W}}}^{2}$ increased to 22% while that of ${\widehat{\upsigma }}_{{\text{Q}}}^{2}$ decreased to 16% (see Additional file 2: Table S5).

Compared to PS mating, with SS mating, the relative standard error of the variance estimates decreased by about 11% for both genetic effects (Table 2 and Fig. 3) and the standard error of the estimated ${{\text{r}}}_{{\text{WQ}}}$ decreased by 8%. For the other genetic parameter sets, the relative reduction in the standard error of ${\widehat{\upsigma }}_{{\text{W}}}^{2}$ and ${\widehat{{\text{r}}}}_{{\text{WQ}}}$ (but not of ${\widehat{\upsigma }}_{{\text{Q}}}^{2}$) was generally greater (see Additional file 2: Tables S4 and S5).

#### Simulating SS or PS controlled mating but estimating genetic parameters and genetic trends with incorrect sire modeling alternatives

**Fig. 4:**
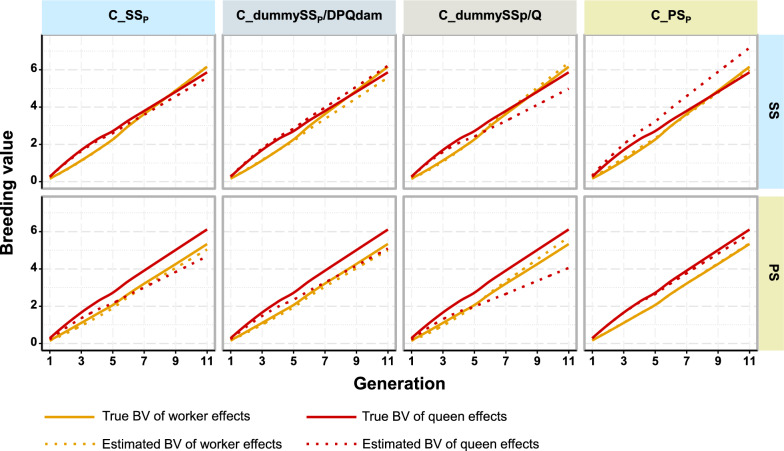
Genetic trends of worker and queen effects under sire modeling scenarios for controlled mating and parameter set 1. The first row of graphs is from scenarios using single sire mating (SS) as the controlled mating strategy in the simulation, and the second row from scenarios using pseudo sire mating (PS). From left to right, results were obtained under sire pedigree modeling scenarios for controlled mating assigning: single sires (C_SS_P_); a dummy single sire per dam of DPQ(s) (C_dummySSp/DPQdam); a dummy single sire for each mating (C_dummySS_P_/Q); and pseudo sires (C_PS_P_)

In this section, the results were obtained assuming that the only known information on a BQ’s mate was the dam of the DPQ(s) with whom she had mated. Thus, it was assumed that it was not known whether the DPQs mated with each BQ jointly, as a PS, or separately, each as a SS. Under this hypothesis, when the dam of the DPQs was known, but it was not known whether DPQs were used as SS or PS, strong biases in the estimated variance(s), the estimated ${{\text{r}}}_{{\text{WQ}}}$, or both were observed for some of the scenarios (Fig. 3). Depending on the scenarios, the relative bias that affected the estimate of the genetic variances ranged from − 32% to + 16%, and the bias that affected ${\widehat{{\text{r}}}}_{{\text{WQ}}}$, ranged from − 0.14 up to + 0.25 (Table 3). Likewise, for the estimated genetic trend (see Fig. 4 and see Additional file 2: Table S3), incorrect mating assumptions led to substantial over or underestimates of the genetic trend of worker and queen effects (from − 13% to + 12% for BV_W_ and from − 39% to + 26% for BV_Q_). More extreme results affecting the estimates of genetic variances, correlations, and trends were obtained for the other parameter sets (see Additional file 2: Tables S3, S4, and S5). Regardless of the genetic parameter set, the strongest relative bias and relative SE that affected the estimates of genetic variances were obtained with PS mating and when a single dummy SS_P_ per DPQ dam was assigned in the pedigree file.

**Table 3:** Errors in genetic parameter estimates when sires for controlled mating were not correctly modeled in the pedigree

Sire pedigree modeling for controlled mating	${\widehat{\upsigma }}_{{\text{W}}}^{2}$			${\widehat{\upsigma }}_{{\text{Q}}}^{2}$			${\widehat{{\text{r}}}}_{{\text{WQ}}}$	
	Relative bias (%)	Relative SE (%)	% strong deviations	Relative bias (%)	Relative SE (%)	% strong deviations	Bias	SE
Controlled mating strategy: SS								
C_dummySS_p_/DPQdam	− 27.03	14.85	66	− 6.67	17.25	28	0.248	0.174
C_dummySS_p_/Q	15.08	25.38	48	2.68	20.06	32	− 0.111	0.168
C_PS_P_	6.58	23.88	44	16.15	21.80	42	0.069	0.169
Controlled mating strategy: PS								
C_SS_P_	− 26.90	14.08	68	− 22.71	14.93	60	0.062	0.166
C_dummySS_p_/DPQdam	− 32.27	12.82	84	− 22.46	15.59	59	0.194	0.176
C_dummySS_p_/Q	3.67	22.25	38	− 14.02	17.32	44	− 0.139	0.180

${\widehat{\upsigma }}_{{\text{W}}}^{2}$, ${\widehat{\upsigma }}_{{\text{Q}}}^{2}$ and ${\widehat{{\text{r}}}}_{{\text{WQ}}}$: estimates of the genetic variances and correlation for worker and queen effect

Strong deviations are those that differ by more than 20% from the true values. The relative bias was calculated as $\left(\frac{1}{{n}_{rep}}\sum_{1}^{{n}_{rep}}\left(\frac{{\widehat{\upsigma }}_{{\text{A}}}^{2}-{\upsigma }_{{\text{A}}}^{2}}{{\upsigma }_{{\text{A}}}^{2}}\right)\right)$. The relative standard error (SE) was the standard deviation across replicates of the relative difference between estimates and the true value

Controlled mating strategy in the simulation: SS (single sire) and PS (pseudo-sire) mating

Sire pedigree modeling for controlled mating: C_SS_P_, C_dummySS_P_/DPQdam, C_dummySS_P_/Q, C_PS_P_: controlled mated queens are assigned, respectively, in the pedigree single sires, dummy single sires per dam of DPQ(s), dummy single sires per mated queen, pseudo-sires

### Simulation Set II: sire modeling for the open mating of drone-producing queens

In simulation Set II, DPQs were mated with a heterogeneous open-mating drone population, simulating two distinct subpopulations that differed in their genetic level.

**Fig. 5:**
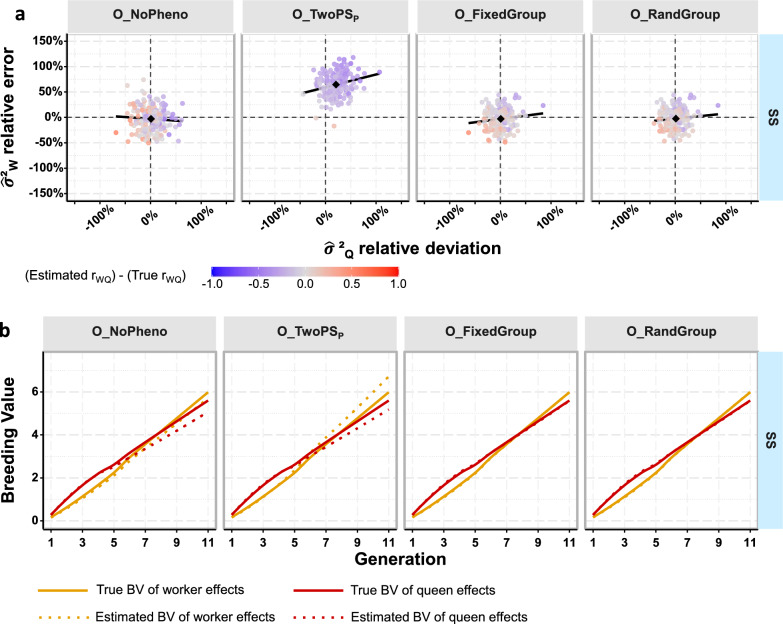
Errors on estimates of genetic parameters (**a**) and breeding values (**b**) under sire modeling scenarios for open mating. Black diamond-shaped points indicate the relative bias on genetic variances, and black lines the regression lines of relative errors of variance estimates of worker effects on that of queen effects. The controlled mating strategy in the simulation always used single sire mating (SS). DPQs were open-mated with a heterogeneous drone population. From left to right, the results were obtained by excluding DPQs colony records (O_NoPheno); accommodating for the effect of subpopulations of open-mating drones by: assigning a distinct open-mating pseudo sire in the pedigree for initial BQs and each half of the DPQs (O_TwoPS_P_); or by adding a fixed (O_FixedGroup) or random (O_RandGroup) non-genetic effect in the statistical model describing the phenotypes, with one level per drone subpopulation

The exclusion of DPQ colony records from the genetic evaluation (O_NoPheno) did not cause any strong bias in the genetic parameter estimates (see Table 4 and Additional file 2: Table S6), although family selection was performed on these colony phenotypes. Nevertheless, excluding these records increased the standard error of the estimated genetic variances (about 19% for both genetic effects), the genetic correlation (33%), and the residual variance (33%), when compared to the scenario that included these records and in which a fixed effect for the drone subpopulations was added in the evaluation model (O_FixedGroup) (see Fig. 5a, and Additional file 2: Table S6). Furthermore, excluding the DPQ colony records also led to an underestimation of the genetic trend of queen effects (− 15%, see Fig. 5b and Additional file 2: Table S7).

**Table 4:** Errors in genetic parameter estimates for sire modeling scenarios relative to open mating

Sire modelling scenario	${\widehat{\upsigma }}_{{\text{W}}}^{2}$			${\widehat{\upsigma }}_{{\text{Q}}}^{2}$			${\widehat{{\text{r}}}}_{{\text{WQ}}}$	
	Relative bias (%)	Relative SE (%)	% strong deviations	Relative bias (%)	Relative SE (%)	% strong deviations	Bias	SE
O_NoPheno	− 3.02	23.09	40	1.04	21.74	34	− 0.021	0.195
O_TwoPS_P_	64.45	22.34	98	21.03	20.75	54	− 0.245	0.110
O_FixedGroup	− 3.04	19.39	34	0.83	18.37	24	0.003	0.146
O_RandGroup	− 2.49	18.91	33	1.24	17.84	23	− 0.003	0.137

Sire modelling scenarios: O_NoPheno: modeling a single PS_P_ and open-mated DPQ’s colony phenotypes excluded; O_TwoPS_P_: modeling one open mating PS_P_ per open mating drone subpopulation; O_FixedGroup: modeling a single PS_P_ and drone subpopulations accounted for by a non-genetic fixed effect; O_RandGroup: modeling a single PS_P_ and drone subpopulations accounted for by a non-genetic random effect

${\widehat{\upsigma }}_{{\text{W}}}^{2}$, ${\widehat{\upsigma }}_{{\text{Q}}}^{2}$ and ${\widehat{{\text{r}}}}_{{\text{WQ}}}$: estimates of the genetic variances and correlation for worker and queen effect

Strong deviations are those that differ by more than 20% from the true values. The relative bias was calculated as $\left(\frac{1}{{n}_{rep}}\sum_{1}^{{n}_{rep}}\left(\frac{{\widehat{\upsigma }}_{{\text{A}}}^{2}-{\upsigma }_{{\text{A}}}^{2}}{{\upsigma }_{{\text{A}}}^{2}}\right)\right)$. The relative standard error (SE) was the standard deviation across replicates of the relative difference between estimates and the true value

Accounting for the open mating of DPQs by modeling a different open-mating PS for each drone subpopulation (O_TwoPS_P_) led to a marked overestimation of the variance of worker effects (+ 64%, Table 4) and, to a lesser extent, that of queen effects (+ 21%). The relative bias for both effects was approximately halved in the scenarios with a doubled variance of worker effects (see Additional file 2: Table S6). These overestimated variances were accompanied by an underestimation of the genetic correlation between worker and queen effects of − 0.25 (see Fig. 5a and Table 4). Regarding genetic trend, it was overestimated for worker effects (+ 17% see Additional file 2: Table S7 for ${{\text{r}}}_{{\text{WQ}}}$ = 0 and ${\upsigma }_{{\text{W}}}^{2}$ = 10), while it was underestimated for queen effects to a similar degree (− 13%) as for the scenario where DPQ records were excluded from the genetic evaluation.

Finally, the results indicated that the best strategy to account for the heterogeneous open-mating drone population was to add a fixed (O_FixedGroup) or a random (O_RandGroup) effect in the evaluation model with one level for each drone subpopulation. This modeling led to little or no bias and the smallest standard errors for the estimates of genetic parameters and genetic trends. The differences between these two approaches were generally minimal (Fig. 5a). The only notable difference concerned the genetic parameter set with equal variances for worker and queen effects and a negative ${{\text{r}}}_{{\text{WQ}}}$ (see Additional file 2: Table S6). In that case, considering the heterogeneity of the mating of DPQs by adding a random non-genetic effect prevented convergence in approximately one quarter of the repetitions. When they converged, the estimates were very similar to those obtained with the fixed effect model.

## Discussion

This study investigated the impact of sire modeling on the estimates of genetic parameters and genetic trends. First, we focused on the controlled mating of BQs and explored the relevance of knowing how the three sister-DPQs of each known dam of DPQs had been used (as SS or PS). Second, we studied the open mating of phenotyped DPQs, accounting for the subpopulations of drones that mate with these queens in three ways: first, by excluding the DPQ phenotypes; second, through the modeling of genetic effects by assuming a separate open-mating PS for each drone subpopulation; and last, by adding a non-genetic effect (fixed or random) in the evaluation model for each drone subpopulation.

We chose to vary neither the number of drones (e.g. from 8 to 16) mating with each queen nor the number of selected DPQs per DPQ dam (e.g. from three to eight), because preliminary trials had shown that they had only a marginal impact on the results. In order to understand the impact of erroneous assumptions, we simulated only systematic errors that affected all matings. Obviously, in real datasets, a combination of erroneous and correct assumptions is likely, thus producing less extreme results.

### Sire modeling for the controlled mating of breeding queens

Our results demonstrate the importance of knowing as precisely as possible how DPQs of a particular dam were used: either separately (SS) or jointly (PS). When used jointly, which DPQs exactly composed the group has not yet been considered in theory [6], since the sisters making up a PS are supposed to be unselected and random progeny of their dam. However, knowledge of this information could be useful when DPQ colony phenotypes are used for the estimation of breeding values. If DPQs were selected from a sister-group to form a PS, considering them as random progeny could create biased estimates. Other approaches have been developed to handle pedigree uncertainty regarding sires [28] that might avoid such biases but have not yet been implemented in honeybees. However, these approaches would need to assign a correct probability to each suspected sire in order to weight the relationship coefficient accordingly, which seems difficult given that the contribution of each DPQ and of each drone [29] to a queen’s offspring is difficult to predict.

#### The most accurate estimates: single sire mating

Knowledge of whether SS mating is used is important because the true pedigree relationships between DPQs and descendants are known in that situation and there is no need to account for them probabilistically. In addition, the phenotypic information on the single DPQ can be better used in the estimation of variance components and breeding values, thus further reducing the standard errors of estimates. This gain in precision was notable (Table 2), although some theoretical flaws existed regarding how SS mating was considered when deriving the relationship matrix (as noted by Manual Du, personal communication). In fact, for SS mating, we simply used the general formulas described by Brascamp and Bijma [6]; in particular, we assumed that the probability of two female offspring coming from the same DPQ (p_2_) followed a Poisson distribution, so that p_2_ $\approx \frac{1}{{\text{D}}}+\frac{1}{{\text{S}}}$, where ${\text{S}}$ is the number of sister-DPQs contributing the ${\text{D}}$ drones mating with a queen. When assuming SS mating in the estimation, ${\text{S}}$ equaled 1, leading to p_2_ > 1. The new version of the software used to derive the inverse relationship matrix was corrected for this, and has also been made available.

#### Estimates of genetic parameters and trends with uncertainty on how DPQs of each dam were used

Apart from the loss in precision when SS was not assumed although it was the true mating strategy, uncertainty regarding the exact way DPQs were used for mating, and subsequent incorrect assumptions in the genetic evaluation, could give rise to strong biases or increased standard errors in the estimates of both the genetic parameters and genetic trends (Table 3 and see Additional file 2: Table S3).

The biases observed can be synthesized and interpreted as follows. On the one hand, if the assumptions of how DPQs were used in mating lead to an average overestimation of the genetic relationship between the offspring of BQs and DPQs, then the genetic variances will be underestimated, while the genetic correlation will be overestimated (Fig. 3). Under our simulations, this occurred when a dummy SS_P_ per dam of DPQs was assumed (when the mating strategy was PS_P_ but also SS_P_). Similarly, but to a lesser degree, the relationship between the offspring of BQs and DPQs was also overestimated when SS_P_ was assumed while the actual mating strategy was PS. In that case, genetic variances were also markedly underestimated but the genetic correlation was much less biased. On the other hand, if the assumptions regarding the use of DPQs during mating lead to an average underestimation of the genetic relationship between the offspring of BQs and DPQs, then the genetic variance estimates will only be moderately biased, but their standard error becomes larger, while the genetic correlation is underestimated. Under our simulations, this occurred when a dummy SS_P_ per mating was assumed (regardless of the mating strategy). Similarly, but to a lesser extent, the genetic relationship between the offspring of BQs and DPQs was also underestimated when PS_P_ was assumed while the mating strategy was SS. In that case, genetic variances were moderately overestimated with intermediate standard errors, and the genetic correlation was only moderately biased.

### Sire modeling for the open mating of drone-producing queens

For open mating, where the drones in each open-mating subpopulation have different mean breeding values, exclusion of DPQ colony records from the analysis did not lead to marked biases in the genetic parameter estimates, although these phenotypes had been used for selection. This held true even if the selection intensity of DPQs was markedly increased (selecting three times less DPQs, data not shown).

Adding a dummy open PS_P_ to the pedigree for each of the two drone subpopulations led to overestimated genetic variances. This suggests that the difference of one genetic standard deviation (SD) between the two subpopulations ended up in the genetic variance estimate. The difference between the two subpopulations, and consequently between the worker groups that they generated, resulted in two sets of DPQ colonies with a systematically different trait value in the data. A difference of one genetic SD between single individuals in the base generation is not unlikely and should not lead to inflated genetic parameter estimates. However, the dummy PS represented a group of 100 individuals, so that the SD of its mean value was much smaller than one genetic SD, as reflected by the small coefficient for the PS on the diagonal of the relationship matrix. Hence, a clear difference between the two PSs could only be explained by a large genetic variation in the base generation, resulting in an overestimation of the genetic variances.

To determine whether increasing the genetic variability of open-mating drones affected the estimates of the genetic parameters and trends, we increased the (co)variance of worker and queen effects fourfold to generate the BVs of these drones, while maintaining the difference in the mean genetic value of the two drone subpopulations. However, the results (not shown) were very similar to those presented in this study.

In this study, open mating with a heterogeneous drone population only concerned DPQs. The mates of DPQs only impacted the phenotypes of DPQ colonies, because they did not contribute genetically to the breeding population, since they were only the sires of the (sterile) worker groups of DPQs. If BQs had also been open-mated to a heterogeneous drone population, then the effects of their mates should be accounted for by genetic effects, as they would contribute fertilized eggs to the breeding nucleus. This could be done using genetic groups [30], but the derivations would require an adaptation to the specific structure of honeybee relationship matrices.

### Effect of the breeding nucleus size on the estimates of genetic parameters and trends

The results obtained with the base genetic parameter set and repeated with 12 and 36 maternal families were very similar in terms of mean estimated (co)variances and genetic trends, with an expected difference of smaller (or larger) standard errors of estimates for larger (or smaller) nucleus size scenarios. These differences regarding the genetic variances were close to what could be approximated from the results obtained with ${\text{N}}$ = 24 maternal families by considering the standard errors as proportional to $\frac{1}{\surd {\text{N}}}$ (see Additional file 4: Fig. S1).

In addition, the standard errors of the estimates that were obtained (calculated from estimated (co)variances over replicates) were in good accordance with the mean predicted standard errors obtained by AIReML (see Additional file 2: Table S8).

### Effect of the true genetic correlation between worker and queen effects

The standard error of the ${{\text{r}}}_{{\text{WQ}}}$ estimate was smaller in the genetic parameter sets with a negative ${{\text{r}}}_{{\text{WQ}}}$ than in those with a null ${{\text{r}}}_{{\text{WQ}}}$. This was consistent with previous findings in simulated honeybee datasets [19] and also agreed with theoretical predictions [31]. For given genetic variances of worker and queen effects, a negative ${{\text{r}}}_{{\text{WQ}}}$ also decreases the phenotypic variance, leading to higher heritability values for the worker and for the queen effects. Another reason for this observation is that we forced the estimated ${{\text{r}}}_{{\text{WQ}}}$ to be within the parameter space of [− 1; 1], resulting in smaller errors for true values nearer to the bounds.

### Practical implications

#### Importance of knowing how DPQs were used in controlled mating

In breeding plans that involve isolated mating stations, a DPQ dam at each station will usually be represented by a group of sister-DPQs. However, in the case of artificial insemination, it is common to use the same dam of DPQs in multiple ways, such as by collecting sperm from a single DPQ or from a few sister-DPQs. However, apart from the dam of DPQs, the usage of DPQs is often not recorded. We demonstrated that the challenges associated with obtaining accurate estimates of genetic parameters and trends in honeybees are exacerbated when we lack information on how DPQs are used. Therefore, if the DPQs were used jointly, as a PS, this should be recorded. With PS mating, the number of sisters composing the group should also be recorded, as unpublished results have shown that this latter parameter will impact the genetic evaluation. If the DPQs were used separately, as a SS, knowing which particular DPQ is used is also important, since assigning dummy DPQs also deteriorates the reliability of genetic estimates.

#### The most reliable estimates for an unknown controlled mating strategy

If information on the use of DPQs in controlled mating cannot be obtained, the estimates of genetic parameters and trends might become severely biased. Nonetheless, if the true mating of BQs is unknown, an assumption must be made as to how DPQs were used in the matings (either as PS_P_ or SS_P_). Our results suggest that assuming DPQs were used jointly, as a PS, produced the highest probabilities of obtaining genetic parameter estimates that do not deviate markedly from their true values. Compared to other erroneous assumptions, assuming PS_P_ gave the most accurate estimates when, of course, PS mating was used in the simulation, but also generally when SS was used (see Additional file 2: Tables S4 and S5).

However, all assumptions other than SS_P_ for SS and PS_P_ for PS led to larger errors in the predicted BVs of either worker or queen effects, or both (Fig. 4 and see Additional file 2: Table S3). Assuming PS_P_ when SS was used led to unbiased estimates of the genetic trend of worker effects, but tended to overestimate genetic gain on queen effects. Although this would deserve more research, we suggest that assuming PS_P_ when the true mating strategy is unknown could also be recommended when the main focus is on estimating genetic trends.

#### Recommended strategy to include phenotypes of open-mated DPQs

In the case of artificial insemination, where the drones used to inseminate a BQ come from a few sister DPQs, their colonies are often phenotyped. These DPQs may be open mated, as is common in France [8] or Italy (personal communication from Melyos Apicoltura), for example. To integrate the phenotypes of DPQs open mated with a heterogeneous drone population, we suggest adding a non-genetic effect in the evaluation model, with one level for each drone subpopulation. These subpopulations could, for example, correspond to different geographic areas and periods in the season (or across years) where the DPQs were mated. With numerous and small drone subpopulations, a random effect may be preferred over a fixed effect. In contrast to assigning an open-mating PS_P_ as the male mate of these DPQs, including a non-genetic effect to account for the two drone subpopulations can avoid bias. Using either a fixed or random non-genetic effect in the evaluation model produced very similar results. However, we modeled a simplistic situation in which all the colonies tested in a year were affected by the same identified environmental effect. Differences between modeling by fixed or random effects might appear if there is statistical confounding between the mean genetic effect of open-mating drones in an apiary and the environmental effects of this apiary, under a design where the genetic connectedness between apiaries is limited.

## Conclusions

When breeding queens are mated with drones that are produced by a single DPQ (as sometimes happens with artificial insemination) and this mating strategy is appropriately modeled, estimates of genetic parameters and genetic trends are more precise than in situations where queens are mated with drones that are produced by a group of sister-DPQs. However, if breeders only record which dam of the DPQ(s) is used, but not which particular DPQ or group of sister-DPQs, erroneous assumptions can lead to marked biases that affect these genetic estimates. When the true mating strategy is unknown, assuming that drones come from a group of sister-DPQs leads to the highest probability of obtaining genetic parameter estimates that do not markedly deviate from their true values. However, marked bias in the estimated genetic trend of queen effects is observed. Moreover, if the DPQs are open-mated with a heterogeneous drone population and their phenotypes are used in the genetic analysis, then we recommend the addition of a non-genetic effect for drone origin in the evaluation model.

### Supplementary Information

**Additional file 1: Text S1.** Infinitesimal model applied to the honeybee. The main equations for the infinitesimal model applied to the honeybee as described by Kistler et al. [20] are shown and describe the generation of base queens and drones, colony phenotype modelling, and lastly BV inheritance for queens, drones, and worker groups.

**Additional file 2: Table S1.** Input parameters for the simulations. The table shows the main input parameters for all the simulations, including genetic parameter sets other than that used for the main text. σ^2^: variance of worker (W), queen (Q) or residual (e) effects in the base population. The only environmental fixed effect was a year effect with variance ${\upsigma }_{{\text{year}}}^{2}$.${{\text{r}}}_{{\text{WQ}}}$: genetic correlation between worker and queen effects. BQs: breeding queens, DPQs: drone-producing queens. **Table S2.** AIReML starting parameter values and convergence criteria. The table shows the initial values used to estimate (co)variances ${\upsigma }_{{\text{W}}}^{2}$, ${\upsigma }_{{\text{Q}}}^{2}$, ${\upsigma }_{{\text{e}}}^{2}$ and ${\upsigma }_{{\text{WQ}}}$, respectively, for the worker, queen and residual effects, and the covariance between worker and queen effects. The true values for genetic variances ${\upsigma }_{{\text{W}}}^{2}$ and ${\upsigma }_{{\text{Q}}}^{2}$ were 10 and 20, respectively, and for the covariance 0, − 5, or approximately − 7, respectively, depending on the genetic parameter set. The true ${\upsigma }_{{\text{e}}}^{2}$ was always equal to 30. **Table S3.** True and estimated genetic trends for all genetic parameter sets and sire modeling scenarios of controlled mating. ${\upsigma }_{{\text{W}}}^{2}$, ${{\text{r}}}_{{\text{WQ}}}$: genetic variance of worker effects and genetic correlation between worker and queen effects. The genetic trends (true and estimated) were calculated as the linear regression coefficients of true breeding values (BV) and estimated breeding values (EBV) for worker (W) and queen (Q) effects over breeding years (after the fifth year of the breeding program, when the nucleus became closed). Controlled mating strategy under the simulation: SS (single sire) and PS (pseudo sire) mating. Sire pedigree modeling for controlled mating: C_SS_P_, C_dummySS_P_/Q, C_dummySS_P_/DPQdam, C_PS_P_: controlled mated queens are assigned respectively in the pedigree single sires, dummy single sires per dam of DPQ(s), dummy single sires per mated queen, pseudo sires. **Table S4.** Errors affecting estimates for genetic parameter sets with a null ${{\text{r}}}_{{\text{WQ}}}$ and all sire modeling scenarios for controlled mating. ${\upsigma }_{{\text{W}}}^{2}$, ${\upsigma }_{{\text{Q}}}^{2}$, ${{\text{r}}}_{{\text{WQ}}}$: genetic variances and correlation for worker and queen effects. ${\upsigma }_{{\text{e}}}^{2}$: residual variance. Estimates are denoted by ‘^’; strong deviations differ by more than 20% from the true values. Controlled mating strategy under the simulation: SS (single sire) and PS (pseudo sire) mating. Sire pedigree modeling for controlled mating: C_SS_P_, C_dummySS_P_/DPQdam, C_dummySS_P_/Q, C_PS_P_: controlled mated queens are assigned respectively in the pedigree single sires, dummy single sires per dam of DPQ(s), dummy single sires per mated queen, pseudo sires. **Table S5.** Errors affecting estimates for genetic parameter sets with a negative ${{\text{r}}}_{{\text{WQ}}}$ and all sire modeling scenarios for controlled mating. ${\upsigma }_{{\text{W}}}^{2}$, ${\upsigma }_{{\text{Q}}}^{2}$, ${{\text{r}}}_{{\text{WQ}}}$: genetic variances and correlation for worker and queen effects. ${\upsigma }_{{\text{e}}}^{2}$: residual variance. Estimates are denoted by ‘^’; strong deviations differ by more than 20% from the true values. Controlled mating strategy under the simulation: SS (single sire) and PS (pseudo sire) mating. Sire pedigree modeling for controlled mating: C_SS_P_, C_dummySS_P_/DPQdam, C_dummySS_P_/Q, C_PS_P_: controlled mated queens are assigned respectively in the pedigree single sires, dummy single sires per dam of DPQ(s), dummy single sires per mated queen, pseudo sires. **Table S6.** Errors affecting estimates for all genetic parameter sets and sire modeling scenarios for open mating. ${\upsigma }_{{\text{W}}}^{2}$, ${\upsigma }_{{\text{Q}}}^{2}$, ${{\text{r}}}_{{\text{WQ}}}$: genetic variances and correlation for worker and queen effects. ${\upsigma }_{{\text{e}}}^{2}$: residual variance. Estimates are denoted by ‘^’; strong deviations differ by more than 20% from the true values. The controlled mating strategy was single sire mating (SS_S_). Sire modeling for open mating: O_NoPheno: open-mated DPQ colony phenotypes are excluded from the genetic analysis; O_PS_P,_ O_TwoPS_P_: open-mated DPQs are assigned in the pedigree respectively a single open mating pseudo sire, or one for each open-mating drone subpopulation (initial BQs and each half of the DPQ); O_FixedGroup or O_RandGroup: the effect of drone subpopulations mating DPQs is accounted for by adding a fixed or a random non-genetic effect in the statistical model that describes the phenotypes. **Table S7.** True and estimated genetic trends for all genetic parameter sets and sire modeling scenarios for open mating. ${\upsigma }_{{\text{W}}}^{2}$, ${{\text{r}}}_{{\text{WQ}}}$: genetic variance of worker effects and genetic correlation between worker and queen effects. The genetic trends (true and estimated) were calculated as the linear regression coefficients of true breeding values (BV) and estimated breeding values (EBV) for worker (W) and queen (Q) effects over breeding years (from the fifth year of the breeding program, when the nucleus became closed). The controlled mating strategy was single sire mating (SS). Sire modeling for open mating: O_NoPheno: open-mated DPQ colony phenotypes are excluded from the genetic analysis; O_PS_P,_ O_TwoPS_P_: open-mated DPQs are assigned in the pedigree respectively a single open mating pseudo sire, or one for each open-mating drone subpopulation (initial BQs and each half of the DPQ); O_FixedGroup or O_RandGroup: the effect of drone subpopulations mating DPQs is accounted for by adding a fixed or a random non-genetic effect in the statistical model that describes the phenotypes. **Table S8.** AIReML predicted and realized standard errors (SE) of genetic (co)variances. Predicted SE: mean prediction (by the inverse averaged information matrix) of the SE of genetic (co)variance estimates over repetitions. Realized SE: SD over repetitions of the error on the variance estimates of worker $({\widehat{\upsigma }}_{{\text{W}}}^{2})$ and queen $({\widehat{\upsigma }}_{{\text{Q}}}^{2})$ effects, as well as the covariance (${\widehat{\upsigma }}_{{\text{WQ}}})$. Controlled mating strategy in the simulation: SS (single sire) and PS (pseudo sire) mating. Sire pedigree modeling for controlled mating: C_SS_P_, C_dummySS_P_/DPQdam, C_dummySS_P_/Q, C_PS_P_: controlled mated queens are assigned respectively in the pedigree single sires, dummy single sires per dam of DPQ(s), dummy single sires per mated queen, pseudo sires.

**Additional file 3: Text S2.** Implementation of open-mating pseudo sires for the estimation of genetic parameters and breeding values. Details are given on how open-mating sires, which are dummy individuals in the pedigree, were modeled, with a description of how their relationship coefficients are calculated in the honeybee specific relationship matrix [6, 21]. A minimalistic example is shown.

**Additional file 4: Figure S1.** Realized and predicted (from 24 family scenario results) standard errors of the genetic variance estimates. The first row of graphs is from scenarios using single sire mating (SS) as the controlled mating strategy in the simulation, and the second row from scenarios using pseudo sire mating (PS). From left to right, results were obtained from sire pedigree modeling scenarios for controlled mating assigning single sires (C_SS_P_); a dummy single sire per dam of DPQs (C_dummySS_P_/DPQdam); a dummy single sire for each mating (C_dummySS_P_/Q); and pseudo sires (C_PS_P_). se (${\upsigma }_{{\text{a}}}^{2}$) is the standard error of estimated genetic variance, for either worker or queen effects (see color legend). Dashed lines link the standard error predicted for N = 12 and N = 36 maternal families, using the values obtained with 24 maternal families, and dividing it by $\sqrt{\frac{{\text{N}}}{2}}$. Continuous lines link standard errors obtained when running simulations with all three breeding nucleus sizes: 12, 24 and 36 maternal families. Simulations used genetic parameter set one, with a null genetic correlation between worker and queen effects and equal variances for both effects, using 200 replicates. The predicted and realized standard errors are similar.

## Data Availability

The R simulation code used during the current study is not publicly available while the authors continue to perform active software developments. They may be available from the corresponding author upon reasonable request. The R code used to obtain the inverse of honeybee-specific relationship matrices is available at the AINV-honeybees GitHub repository https://github.com/Tristan-Kistler/AINV-honeybees/ and archived on Zenodo: 10.5281/zenodo.7951334. The version used in this study was v19. Further developments will also be available via the GitHub repository.
