# Supplementary material for: Uncertainty in the mating strategy of honeybees causes bias and unreliability in the estimates of genetic parameters
Source: Genet Sel Evol. 2024 Apr 17;56:30. doi: 10.1186/s12711-024-00898-3 (PMC11022492; doi:10.1186/s12711-024-00898-3)
Supplement: Supplementary file 1 — Additional file 1: Text S1. Infinitesimal model applied to the honeybee. The main equations for the infinitesimal model applied to the honeybee as described by Kistler et al. [20] are shown and describe the generation of base queens and drones, colony phenotype modelling, and lastly BV inheritance for queens, drones, and worker groups. [file 12711_2024_898_MOESM1_ESM.docx]

**Additional file 1 Text S1**

**Infinitesimal model applied to the honeybee**

Generation of base individuals:
$\mathbf{B}\mathbf{V}^{\mathbf{base} \mathbf{BQs}}\boldsymbol{\sim}\boldsymbol{N}\boldsymbol{(}\boldsymbol{0}\boldsymbol{,}\boldsymbol{\Sigma}_{\mathbf{BV}}^{\mathbf{2}}\boldsymbol{)}$: Breeding queens (BQ) from the base population had their **BV** drawn from a bivariate normal distribution centered on **0** and with (co)variance matrix $\boldsymbol{\Sigma}_{\mathbf{BV}}^{\mathbf{2}}$.
$\mathbf{B}\mathbf{V}^{\mathbf{base} \mathbf{Ds}}\boldsymbol{\sim}\boldsymbol{N}\boldsymbol{(}\boldsymbol{0}\boldsymbol{,}\boldsymbol{\Sigma}_{\mathbf{BV}}^{\mathbf{2}}\boldsymbol{)}$: Base drones, which are haploid, had their **BV** (co)varied half as much as BQs. In simulation set I, open mating drones mating DPQs had their **BV** centered on **0** (unstructured open mating drone population), while in simulation set II, one half of the drones mating DPQs had their **BV** centered on $\boldsymbol{-}\boldsymbol{\alpha}$ and the other half on $+\boldsymbol{\alpha}$ **(**structured open mating drone population).

After mating, queens produced offspring worker groups by:

$\overline{\mathbf{B}\mathbf{V}^{\mathbf{W}}}=\frac{1}{2}\cdot\mathbf{B}\mathbf{V}^{\mathbf{Q}\mathbf{ueen}}+\overline{\mathbf{B}\mathbf{V}^{\mathbf{D}\mathbf{rone}\mathbf{s}}}$

where the breeding value of a worker group ($\overline{\mathbf{B}\mathbf{V}^{\mathbf{W}}}$) equaled the sum of half the **BV** of its BQ and the average **BV** of the drones that mated the BQ.

Colonies’ phenotypes were obtained using:

$Perf=BV_{\mathrm{mat}}^{\mathrm{Queen}}+ \frac{1}{2}BV_{\mathrm{dir}}^{\mathrm{Queen}}+\overline{BV_{\mathrm{dir}}^{\mathrm{Drones}}}$

where the performance (Perf) of a colony equaled the sum of the maternal genetic effect of the queen $\left( BV_{\mathrm{mat}}^{\mathrm{Queen}} \right)$, half the direct genetic effect of the queen $\left( \frac{1}{2}BV_{\mathrm{dir}}^{\mathrm{Queen}} \right)$ and the average direct genetic effect of the drones that mated the queen.

Offspring queens inherited their BVs following:

$\mathbf{B}\mathbf{V}^{\mathbf{Q}\mathbf{ueen}}=\frac{1}{2}\cdot\mathbf{B}\mathbf{V}^{\mathbf{BQ}}\mathbf{+}\mathbf{B}\mathbf{V}^{\mathbf{D}\mathbf{rone}}+\boldsymbol{\varphi}^{\mathbf{BQ}}$

where their **BV** was the sum of half their BQ’s **BV**, the **BV** of one of the drones that mated their BQ, and a mendelian sampling term (**φ**) drawn from $\mathbf{N(}\mathbf{0}\mathbf{,}\frac{1}{4}\cdot(1-F)\cdot\boldsymbol{\Sigma}_{\mathbf{BV}}^{\mathbf{2}}\boldsymbol{)}$, where F is the inbreeding coefficient of the offspring’s BQ.

Lastly, drones inherited their BVs following:

$\mathbf{B}\mathbf{V}^{\mathbf{D}\mathbf{rone}}=\frac{1}{2}\mathbf{B}\mathbf{V}^{\mathbf{DPQ}}+\boldsymbol{\varphi}^{\mathbf{DPQ}}$ (6)

where haploid drones’ **BV** was inherited only from their drone-producing queen (DPQ).

Further details are given in Kistler et al. [20].
