# Supplementary material for: Uncertainty in the mating strategy of honeybees causes bias and unreliability in the estimates of genetic parameters
Source: Genet Sel Evol. 2024 Apr 17;56:30. doi: 10.1186/s12711-024-00898-3 (PMC11022492; doi:10.1186/s12711-024-00898-3)
Supplement: Supplementary file 2 — Additional file 2: Table S1. Input parameters for the simulations. The table shows the main input parameters for all the simulations, including genetic parameter sets other than that used for the main text. σ2: variance of worker (W), queen (Q) or residual (e) effects in the base population. The only environmental fixed effect was a year effect with variance ${\upsigma }_{{\text{year}}}^{2}$σyear2.${{\text{r}}}_{{\text{WQ}}}$rWQ: genetic correlation between worker and queen effects. BQs: breeding queens, DPQs: drone-producing queens. Table S2. AIReML starting parameter values and convergence criteria. The table shows the initial values used to estimate (co)variances ${\upsigma }_{{\text{W}}}^{2}$σW2, ${\upsigma }_{{\text{Q}}}^{2}$σQ2, ${\upsigma }_{{\text{e}}}^{2}$σe2 a [file 12711_2024_898_MOESM2_ESM.docx]

**Additional file 2**

**Table S1 Input parameters for the simulations**

| **Genetic  parameter set** | | | | $\boldsymbol{\sigma}_{\mathbf{e}}^{\mathbf{2}}$ | $\boldsymbol{\sigma}_{\mathbf{year}}^{\mathbf{2}}$ | **Generation interval  (years)** | | **Nb of  maternal families** | **Avg nb of BQs per  family** | **Avg nb of  DPQs per family** | **Number of drones mating a queen** |
| --- | --- | --- | --- | --- | --- | --- | --- | --- | --- | --- | --- |
| **Setup  number** | $\boldsymbol{\sigma}_{\mathbf{W}}^{\mathbf{2}}$ | $\boldsymbol{\sigma}_{\mathbf{Q}}^{\mathbf{2}}$ | $\mathbf{r}_{\mathbf{WQ}}$ |  |  | **Dams** | **Sires** |  |  |  |  |
| 1 | 10 | 10 | 0  0 | 30 | 20 | 1 | 2 | 24 | 18 | 15 | 8 |
| 2 | 20 | 10 |  |  |  |  |  |  |  |  |  |
| 3 | 10 | 10 | -0.5 |  |  |  |  |  |  |  |  |
| 4 | 20 | 10 | -0.5 |  |  |  |  |  |  |  |  |

σ²: variance of either worker (W), queen (Q) or residual (e) effects in the base population. The only environmental fixed effect was a year effect with variance σ²_year_.
r_WQ_: genetic correlation between worker and queen effects.
BQs: breeding queens, DPQs: drone-producing queens.

**Table S2 AIReML starting parameter values and convergence criteria**

| $\boldsymbol{\sigma}_{\mathbf{W}}^{\mathbf{2}}$ | $\boldsymbol{\sigma}_{\mathbf{Q}}^{\mathbf{2}}$ | $\boldsymbol{\sigma}_{\mathbf{WQ}}$ | $\boldsymbol{\sigma}_{\mathbf{e}}^{\mathbf{2}}$ | **Convergence  criterion** | **Maximum  convergence rounds** | **Variance of the additional  random effect for  open mating drone populations (for O_FixedGroup or O_RandGroup)** | **Total nb of phenotyping records in each genetic analysis** |
| --- | --- | --- | --- | --- | --- | --- | --- |
| 15 | 15 | 0.01 | 20 | 1.00E-12 | 100 | 2 | 8,352 |

The table gives the initial values to estimate (co)variances σ²_W_, σ²_Q_, σ²e and σ_WQ_ respectively for worker, queen and residual effects, and the covariance between worker and queen effects. The true values for genetic variances σ²_W_ and σ²_Q_ were respectively 10 and 20, and for the covariance, respectively 0, -5 or approximately -7, depending on the genetic parameter set. The true σ²_e_ was always equal to 30.

**Table S3** **True and estimated genetic trends for all genetic parameter sets and sire modeling scenarios for controlled mating**

| **Simulation scenario** | | | **Sire pedigree modeling for controlled mating** | **Genetic trend for worker effects** | | **Genetic trend for queen effects** | |
| --- | --- | --- | --- | --- | --- | --- | --- |
|  |  |  |  |  |  |  |  |
| $\mathbf{r}_{\mathbf{WQ}}$ | $\boldsymbol{\sigma}_{\mathbf{W}}^{\mathbf{2}}$ | **Controlled mating strategy** |  | **True value** | **Estimate** | **True value** | **Estimate** |
| 0 | 10 | SS | C_SS_P_ | 0.62 | 0.62 | 0.50 | 0.47 |
|  |  |  | C_dummySS_p_/DPQdam | 0.62 | 0.54 | 0.50 | 0.53 |
|  |  |  | C_dummySS_p_/Q | 0.62 | 0.65 | 0.50 | 0.41 |
|  |  |  | C_PS_P_ | 0.62 | 0.59 | 0.50 | 0.63 |
|  |  | PS | C_SS_P_ | 0.52 | 0.50 | 0.54 | 0.40 |
|  |  |  | C_dummySS_p_/DPQdam | 0.52 | 0.49 | 0.54 | 0.43 |
|  |  |  | C_dummySS_p_/Q | 0.52 | 0.58 | 0.54 | 0.33 |
|  |  |  | C_PS_P_ | 0.52 | 0.53 | 0.54 | 0.51 |
|  | 20 | SS | C_SS_P_ | 1.13 | 1.14 | 0.44 | 0.43 |
|  |  |  | C_dummySS_p_/DPQdam | 1.13 | 1.01 | 0.44 | 0.55 |
|  |  |  | C_dummySS_p_/Q | 1.13 | 1.18 | 0.44 | 0.34 |
|  |  |  | C_PS_P_ | 1.13 | 1.09 | 0.44 | 0.61 |
|  |  | PS | C_SS_P_ | 0.97 | 0.90 | 0.49 | 0.35 |
|  |  |  | C_dummySS_p_/DPQdam | 0.97 | 0.90 | 0.49 | 0.42 |
|  |  |  | C_dummySS_p_/Q | 0.97 | 1.04 | 0.49 | 0.25 |
|  |  |  | C_PS_P_ | 0.97 | 0.97 | 0.49 | 0.46 |
| -0.5 | 10 | SS | C_SS_P_ | 0.38 | 0.40 | 0.24 | 0.21 |
|  |  |  | C_dummySS_p_/DPQdam | 0.38 | 0.32 | 0.24 | 0.27 |
|  |  |  | C_dummySS_p_/Q | 0.38 | 0.43 | 0.24 | 0.16 |
|  |  |  | C_PS_P_ | 0.38 | 0.34 | 0.24 | 0.35 |
|  |  | PS | C_SS_P_ | 0.27 | 0.28 | 0.33 | 0.21 |
|  |  |  | C_dummySS_p_/DPQdam | 0.27 | 0.26 | 0.33 | 0.22 |
|  |  |  | C_dummySS_p_/Q | 0.27 | 0.36 | 0.33 | 0.14 |
|  |  |  | C_PS_P_ | 0.27 | 0.28 | 0.33 | 0.29 |
|  | 20 | SS | C_SS_P_ | 0.90 | 0.89 | 0.07 | 0.06 |
|  |  |  | C_dummySS_p_/DPQdam | 0.90 | 0.76 | 0.07 | 0.17 |
|  |  |  | C_dummySS_p_/Q | 0.90 | 0.94 | 0.07 | -0.01 |
|  |  |  | C_PS_P_ | 0.90 | 0.82 | 0.07 | 0.21 |
|  |  | PS | C_SS_P_ | 0.69 | 0.67 | 0.18 | 0.10 |
|  |  |  | C_dummySS_p_/DPQdam | 0.69 | 0.66 | 0.18 | 0.12 |
|  |  |  | C_dummySS_p_/Q | 0.69 | 0.80 | 0.18 | -0.01 |
|  |  |  | C_PS_P_ | 0.69 | 0.70 | 0.18 | 0.16 |

σ²_W_, r_WQ_: genetic variance of worker effects and genetic correlation between worker and queen effect.
The annual genetic trends (true and estimate) were calculated as the linear regression coefficients of average true breeding values (BVs) and estimated breeding values (EBV) of BQs for worker (W) and queen (Q) effects over breeding years (after the fifth year of the breeding program, when the nucleus became closed).
Controlled mating strategy in the simulation: SS (single sire) and PS (pseudo sire) mating.
Sire pedigree modeling for controlled mating: C _SS_P_, C_dummySS_P_/DPQdam, C_dummySS_P_/Q , C_PS_P_: controlled mated queens are assigned respectively in the pedigree single sires, dummy single sires per dam of DPQ(s), dummy single sires per mated queen, pseudo sires.

**Table S4 Errors on estimates for genetic parameter sets with a null r_WQ_ and all sire modeling scenarios for controlled mating**

| **Simulation scenario** | **Estimation scenario** | **Estimates** | | | | | | | | | |
| --- | --- | --- | --- | --- | --- | --- | --- | --- | --- | --- | --- |
|  |  | ${\hat{\boldsymbol{\sigma}}}_{\mathbf{e}}^{\mathbf{2}}$ | | ${\hat{\boldsymbol{\sigma}}}_{\mathbf{W}}^{\mathbf{2}}$ | | | ${\hat{\boldsymbol{\sigma}}}_{\mathbf{WQ}}^{\mathbf{2}}$ | | | ${\hat{\mathbf{r}}}_{\mathbf{WQ}}$ | |
|  |  | **Relative bias (%)** | **Relative SE (%)** | **Relative bias (%)** | **Relative SE (%)** | **% strong deviations** | **Relative bias (%)** | **Relative SE (%)** | **% strong deviations** | **Bias** | **SE** |
| **Genetic parameter set 1 (**$\boldsymbol{\sigma}_{\mathbf{W}}^{\mathbf{2}}$ **= 10,** $\boldsymbol{\sigma}_{\mathbf{Q}}^{\mathbf{2}}$ **= 10,** $\mathbf{r}_{\mathbf{WQ}}$**= 0)** | | | | | | | | | | | |
| SS | C_SS_P_ | 1.08 | 3.47 | -0.81 | 18.46 | 27 | -3.45 | 17.50 | 27 | 0.011 | 0.152 |
|  | C_dummySS_p_/DPQdam | 1.18 | 3.71 | -27.03 | 14.85 | 66 | -6.67 | 17.25 | 28 | 0.248 | 0.174 |
|  | C_dummySS_p_/Q | 0.88 | 3.60 | 15.08 | 25.38 | 48 | 2.68 | 20.06 | 32 | -0.111 | 0.168 |
|  | C_PS_P_ | -5.97 | 4.52 | 6.58 | 23.88 | 44 | 16.15 | 21.80 | 42 | 0.069 | 0.169 |
| PS | C_SS_P_ | 7.86 | 3.12 | -26.90 | 14.08 | 68 | -22.71 | 14.93 | 60 | 0.062 | 0.166 |
|  | C_dummySS_p_/DPQdam | 6.65 | 3.34 | -32.27 | 12.82 | 84 | -22.46 | 15.59 | 59 | 0.194 | 0.176 |
|  | C_dummySS_p_/Q | 6.31 | 3.23 | 3.67 | 22.25 | 38 | -14.02 | 17.32 | 44 | -0.139 | 0.180 |
|  | C_PS_P_ | 0.58 | 4.11 | 0.20 | 20.79 | 37 | -2.21 | 19.55 | 30 | 0.013 | 0.165 |
| **Genetic parameter set 2 (**$\boldsymbol{\sigma}_{\mathbf{W}}^{\mathbf{2}}$ **= 20,** $\boldsymbol{\sigma}_{\mathbf{Q}}^{\mathbf{2}}$ **= 10,** $\mathbf{r}_{\mathbf{WQ}}$**= 0)** | | | | | | | | | | | |
| SS | C_SS_P_ | 1.10 | 4.09 | -2.33 | 13.16 | 12 | -3.56 | 17.32 | 30 | 0.017 | 0.130 |
|  | C_dummySS_p_/DPQdam | 1.75 | 4.40 | -24.10 | 12.19 | 64 | -12.49 | 17.01 | 34 | 0.324 | 0.147 |
|  | C_dummySS_p_/Q | 0.68 | 4.22 | 10.20 | 17.85 | 30 | 7.66 | 19.36 | 28 | -0.135 | 0.150 |
|  | C_PS_P_ | -6.70 | 5.39 | 4.78 | 17.93 | 28 | 13.48 | 21.36 | 45 | 0.105 | 0.158 |
| PS | C_SS_P_ | 9.90 | 3.92 | -29.06 | 11.07 | 80 | -25.03 | 15.40 | 62 | 0.084 | 0.162 |
|  | C_dummySS_p_/DPQdam | 7.73 | 4.42 | -29.95 | 11.49 | 80 | -27.50 | 15.64 | 68 | 0.255 | 0.179 |
|  | C_dummySS_p_/Q | 6.69 | 4.04 | -1.32 | 15.17 | 19 | -7.51 | 18.47 | 36 | -0.185 | 0.153 |
|  | C_PS_P_ | 0.70 | 5.28 | -0.31 | 17.42 | 26 | -2.89 | 20.42 | 35 | 0.011 | 0.167 |

$\sigma_{W}^{2}$, $\sigma_{Q}^{2}$, $r_{\mathrm{WQ}}$: genetic variances and correlation for worker and queen effect. $\sigma_{e}^{2}$: residual variance. Estimates are denoted by ’^’; strong deviates differ by more than 20% from the true values.

Controlled mating strategy in the simulation: SS (single sire) and PS (pseudo sire) mating.
Sire pedigree modeling for controlled mating: C _SS_P_, C_dummySS_P_/DPQdam, C_dummySS_P_/Q , C_PS_P_: controlled mated queens are assigned respectively in the pedigree single sires, dummy single sires per dam of DPQ(s), dummy single sires per mated queen, pseudo sires.

**Table S5 Errors on estimates for genetic parameter sets with a negative r_WQ_ and all sire modeling scenarios for controlled mating**

| **Simulation scenario** | **Estimation scenario** | **Estimates** | | | | | | | | | |
| --- | --- | --- | --- | --- | --- | --- | --- | --- | --- | --- | --- |
|  |  | ${\hat{\boldsymbol{\sigma}}}_{\mathbf{e}}^{\mathbf{2}}$ | | ${\hat{\boldsymbol{\sigma}}}_{\mathbf{W}}^{\mathbf{2}}$ | | | ${\hat{\boldsymbol{\sigma}}}_{\mathbf{WQ}}^{\mathbf{2}}$ | | | ${\hat{\mathbf{r}}}_{\mathbf{WQ}}$ | |
|  |  | **Relative bias (%)** | **Relative SE (%)** | **Relative bias (%)** | **Relative SE (%)** | **% strong deviations** | **Relative bias (%)** | **Relative SE (%)** | **% strong deviations** | **Bias** | **SE** |
| **Genetic parameter set 3 (**$\boldsymbol{\sigma}_{\mathbf{W}}^{\mathbf{2}}$ **= 10,** $\boldsymbol{\sigma}_{\mathbf{Q}}^{\mathbf{2}}$ **= 10,** $\mathbf{r}_{\mathbf{WQ}}$**= -0.5)** | | | | | | | | | | | |
| SS | C_SS_P_ | 0.28 | 2.81 | 0.45 | 18.57 | 26 | 0.26 | 15.84 | 18 | -0.017 | 0.099 |
|  | C_dummySS_p_/DPQdam | 1.65 | 2.88 | -33.35 | 16.55 | 81 | -12.98 | 15.58 | 34 | 0.156 | 0.142 |
|  | C_dummySS_p_/Q | -0.32 | 2.94 | 17.89 | 28.17 | 55 | 7.09 | 18.70 | 28 | -0.074 | 0.107 |
|  | C_PS_P_ | -3.16 | 3.42 | 9.32 | 27.67 | 44 | 10.31 | 19.34 | 33 | 0.054 | 0.118 |
| PS | C_SS_P_ | 5.25 | 2.34 | -36.10 | 16.38 | 82 | -25.89 | 13.01 | 68 | 0.063 | 0.136 |
|  | C_dummySS_p_/DPQdam | 4.89 | 2.37 | -40.04 | 15.47 | 90 | -24.79 | 14.15 | 62 | 0.105 | 0.146 |
|  | C_dummySS_p_/Q | 3.40 | 2.39 | -1.78 | 26.23 | 49 | -10.57 | 16.33 | 34 | -0.078 | 0.116 |
|  | C_PS_P_ | 0.90 | 2.77 | -1.01 | 26.13 | 45 | -4.07 | 17.00 | 27 | 0.008 | 0.121 |
| **Genetic parameter set 4 (**$\boldsymbol{\sigma}_{\mathbf{W}}^{\mathbf{2}}$ **= 20,** $\boldsymbol{\sigma}_{\mathbf{Q}}^{\mathbf{2}}$ **= 10,** $\mathbf{r}_{\mathbf{WQ}}$**= -0.5)** | | | | | | | | | | | |
| SS | C_SS_P_ | 0.03 | 3.07 | -0.67 | 14.38 | 14 | -0.53 | 18.79 | 30 | -0.002 | 0.092 |
|  | C_dummySS_p_/DPQdam | 2.62 | 3.21 | -29.96 | 12.27 | 76 | -24.95 | 17.14 | 64 | 0.259 | 0.157 |
|  | C_dummySS_p_/Q | -1.04 | 3.28 | 10.88 | 19 | 34 | 11.38 | 22.72 | 44 | -0.074 | 0.096 |
|  | C_PS_P_ | -2.77 | 3.84 | 8.41 | 19.69 | 34 | 4.40 | 22.82 | 39 | 0.082 | 0.127 |
| PS | C_SS_P_ | 5.85 | 2.72 | -37.40 | 11.58 | 94 | -29.17 | 14.24 | 74 | 0.103 | 0.122 |
|  | C_dummySS_p_/DPQdam | 5.36 | 2.83 | -37.16 | 12.04 | 92 | -31.37 | 15.81 | 78 | 0.176 | 0.147 |
|  | C_dummySS_p_/Q | 2.72 | 2.78 | -8.54 | 16.23 | 29 | -6.96 | 18.43 | 34 | -0.076 | 0.099 |
|  | C_PS_P_ | 0.70 | 3.30 | -0.38 | 19.75 | 34 | -3.47 | 20.68 | 38 | 0.012 | 0.117 |

$\sigma_{W}^{2}$, $\sigma_{Q}^{2}$, $r_{\mathrm{WQ}}$: genetic variances and correlation for worker and queen effect. $\sigma_{e}^{2}$: residual variance. Estimates are denoted by ’^’; strong deviates differ by more than 20% from the true values.
Controlled mating strategy in the simulation: SS (single sire) and PS (pseudo sire) mating.
Sire pedigree modeling for controlled mating: C _SS_P_, C_dummySS_P_/DPQdam, C_dummySS_P_/Q , C_PS_P_: controlled mated queens are assigned respectively in the pedigree single sires, dummy single sires per dam of DPQ(s), dummy single sires per mated queen, pseudo sires.

**Table S6 Errors on estimates for all genetic parameter sets and sire modeling scenarios for open mating**

| **Simulation scenario** | | **Estimation scenario** | **Estimates** | | | | | | | | | | |  |
| --- | --- | --- | --- | --- | --- | --- | --- | --- | --- | --- | --- | --- | --- | --- |
| $\mathbf{r}_{\mathbf{WQ}}$ | $\boldsymbol{\sigma}_{\mathbf{W}}^{\mathbf{2}}$ | **Sire pedigree modeling for open mating** | **convergence (%)** | ${\hat{\boldsymbol{\sigma}}}_{\mathbf{e}}^{\mathbf{2}}$ | | ${\hat{\boldsymbol{\sigma}}}_{\mathbf{W}}^{\mathbf{2}}$ | | | ${\hat{\boldsymbol{\sigma}}}_{\mathbf{WQ}}^{\mathbf{2}}$ | | | ${\hat{\mathbf{r}}}_{\mathbf{WQ}}$ | | |
|  |  |  |  | **Relative bias (%)** | **Relative SE (%)** | **Relative bias (%)** | **Relative SE (%)** | **% strong deviates** | **Relative bias (%)** | **Relative SE (%)** | **% strong deviates** | **Bias** | **SE** | |
| 0 | 10 | O_NoPheno | 100 | 0.80 | 5.09 | -3.02 | 23.09 | 40 | 1.04 | 21.74 | 34 | -0.021 | 0.195 | |
|  |  | O_TwoPS_P_ | 100 | -1.31 | 3.87 | 64.45 | 22.34 | 98 | 21.03 | 20.75 | 54 | -0.245 | 0.110 | |
|  |  | O_FixedGroup | 100 | 0.51 | 3.82 | -3.04 | 19.39 | 34 | 0.83 | 18.37 | 24 | 0.003 | 0.146 | |
|  |  | O_RandGroup | 98 | 0.44 | 3.75 | -2.49 | 18.91 | 33 | 1.24 | 17.84 | 23 | -0.003 | 0.137 | |
|  | 20 | O_NoPheno | 100 | 0.80 | 5.52 | 0.48 | 16.03 | 21 | -2.20 | 25.70 | 47 | -0.013 | 0.189 | |
|  |  | O_TwoPS_P_ | 100 | -0.12 | 4.15 | 28.86 | 14.14 | 68 | 6.99 | 21.82 | 38 | -0.149 | 0.130 | |
|  |  | O_FixedGroup | 100 | 1.02 | 4.24 | -0.30 | 13.77 | 14 | -3.99 | 20.11 | 32 | 0.004 | 0.148 | |
|  |  | O_RandGroup | 100 | 1.03 | 4.23 | -0.33 | 13.75 | 14 | -4.01 | 20.10 | 32 | 0.004 | 0.148 | |
| -0.5 | 10 | O_NoPheno | 100 | 0.26 | 3.55 | 0.75 | 22.54 | 36 | -2.00 | 20.58 | 36 | 0.001 | 0.147 | |
|  |  | O_TwoPS_P_ | 100 | -1.81 | 2.94 | 78.66 | 24.48 | 100 | 31.00 | 20.63 | 74 | -0.145 | 0.073 | |
|  |  | O_FixedGroup | 100 | 0.38 | 2.86 | 0.03 | 21.15 | 32 | -1.14 | 17.67 | 27 | 0.005 | 0.115 | |
|  |  | O_RandGroup | 76 | 0.10 | 2.76 | 5.69 | 19.57 | 29 | -0.83 | 18.17 | 28 | 0.016 | 0.116 | |
|  | 20 | O_NoPheno | 100 | 0.54 | 4.20 | -1.52 | 16.47 | 20 | -2.31 | 22.09 | 36 | -0.014 | 0.132 | |
|  |  | O_TwoPS_P_ | 100 | -0.96 | 2.98 | 32.36 | 15.08 | 78 | 17.77 | 21.08 | 46 | -0.099 | 0.078 | |
|  |  | O_FixedGroup | 100 | 0.65 | 3.04 | -2.13 | 14.68 | 15 | -3.21 | 19.13 | 34 | 0.003 | 0.102 | |
|  |  | O_RandGroup | 100 | 0.65 | 3.04 | -2.14 | 14.67 | 15 | -3.21 | 19.11 | 34 | 0.003 | 0.102 | |

$\sigma_{W}^{2}$, $\sigma_{Q}^{2}$, $r_{\mathrm{WQ}}$: genetic variances and correlation for worker and queen effect. $\sigma_{e}^{2}$: residual variance. Estimates are denoted by ’^’; strong deviates differ by more than 20% from the true values.
The controlled mating strategy was single sire mating (SS).
Sire modeling for open mating: O_NoPheno: open-mated DPQs’ colony phenotypes are excluded from the genetic analysis; O_PS_P,_ O_TwoPS_P_: open-mated DPQs are assigned in the pedigree respectively a single open mating pseudo sire, or one for each open-mating drone subpopulation (initial BQs and each half of the DPQ); O_FixedGroup or O_RandGroup: the effect of the drone subpopulations mating DPQs is accounted for by adding a fixed or a random non-genetic effect in the statistical model describing phenotypes.

**Table S7** **True and estimated genetic trends for all genetic parameter sets and sire modeling scenarios for open mating**

| $\mathbf{r}_{\mathbf{WQ}}$ | $\boldsymbol{\sigma}_{\mathbf{W}}^{\mathbf{2}}$ | **Sire pedigree modeling for open mating** | **Genetic trend for worker effects** | | **Genetic trend for queen effects** | |
| --- | --- | --- | --- | --- | --- | --- |
|  |  |  |  |  |  |  |
|  |  |  | **True value** | **Estimate** | **True value** | **Estimate** |
| 0 | 10 | O_NoPheno | 0.59 | 0.57 | 0.47 | 0.40 |
|  |  | O_TwoPS_P_ | 0.59 | 0.69 | 0.47 | 0.41 |
|  |  | O_FixedGroup | 0.59 | 0.60 | 0.47 | 0.46 |
|  |  | O_RandGroup | 0.59 | 0.60 | 0.48 | 0.46 |
|  | 20 | O_NoPheno | 1.14 | 1.10 | 0.42 | 0.35 |
|  |  | O_TwoPS_P_ | 1.14 | 1.21 | 0.42 | 0.36 |
|  |  | O_FixedGroup | 1.14 | 1.13 | 0.42 | 0.41 |
|  |  | O_RandGroup | 1.14 | 1.13 | 0.42 | 0.41 |
| -0.5 | 10 | O_NoPheno | 0.40 | 0.41 | 0.21 | 0.13 |
|  |  | O_TwoPS_P_ | 0.40 | 0.49 | 0.21 | 0.15 |
|  |  | O_FixedGroup | 0.40 | 0.41 | 0.21 | 0.18 |
|  |  | O_RandGroup | 0.42 | 0.47 | 0.20 | 0.18 |
|  | 20 | O_NoPheno | 0.88 | 0.87 | 0.07 | -0.01 |
|  |  | O_TwoPS_P_ | 0.88 | 0.95 | 0.07 | 0.01 |
|  |  | O_FixedGroup | 0.88 | 0.88 | 0.07 | 0.04 |
|  |  | O_RandGroup | 0.88 | 0.88 | 0.07 | 0.05 |

$\sigma_{W}^{2}$, $r_{\mathrm{WQ}}$: genetic variance of worker effects and genetic correlation between worker and queen effect.
The annual genetic trends (true and estimate) were calculated as the linear regression coefficients of average true breeding values (BVs) and estimated breeding values (EBV) of BQs for worker (W) and queen (Q) effects over breeding years (after the fifth year of the breeding program, when the nucleus became closed).

The controlled mating strategy was single sire mating (SS).
Sire modeling for open mating: O_NoPheno: open-mated DPQs’ colony phenotypes are excluded from the genetic analysis; O_PS_P,_ O_TwoPS_P_: open-mated DPQs are assigned in the pedigree respectively a single open mating pseudo sire, or one for each open-mating drone subpopulation (initial BQs and each half of the DPQ); O_FixedGroup or O_RandGroup: the effect of the drone subpopulations mating DPQs is accounted for by adding a fixed or a random non-genetic effect in the statistical model describing phenotypes.

**Table S8** **AIReML predicted and realized standard errors (SE) of genetic (co)variances**

| **Controlled mating  strategy** | **Sire pedigree modeling for controlled mating** | **SE(**$\boldsymbol{\sigma}_{\mathbf{W}}^{\mathbf{2}}$**)** | | **SE(**$\boldsymbol{\sigma}_{\mathbf{Q}}^{\mathbf{2}}$**)** | | **SE(**$\boldsymbol{\sigma}_{\mathbf{WQ}}$**)** | | |
| --- | --- | --- | --- | --- | --- | --- | --- | --- |
|  |  | **Predicted** | **Realized** | **Predicted** | **Realized** | **Predicted** | **Realized** | |
| **Nucleus size: 12 Breeding Queens** | | | | | | | | |
| SS | C_SS_P_ | 2.75 | 2.65 | 2.44 | 2.56 | 2.07 | 1.93 | |
|  | C_dummySS_p_/DPQdam | 2.25 | 2.18 | 2.43 | 2.57 | 1.89 | 1.75 | |
|  | C_dummySS_p_/Q | 3.72 | 3.71 | 2.75 | 2.79 | 2.76 | 2.78 | |
|  | C_PS_P_ | 3.49 | 3.33 | 3.04 | 3.23 | 2.56 | 2.35 | |
| PS | C_SS_P_ | 2.28 | 2.28 | 2.07 | 1.99 | 1.75 | 1.71 | |
|  | C_dummySS_p_/DPQdam | 2.20 | 2.13 | 2.21 | 2.14 | 1.80 | 1.84 | |
|  | C_dummySS_p_/Q | 3.45 | 3.41 | 2.44 | 2.50 | 2.51 | 2.71 | |
|  | C_PS_P_ | 3.43 | 3.44 | 2.75 | 2.79 | 2.47 | 2.53 | |
| **Nucleus size: 24 Breeding Queens** | | | | | | | |  |
| SS | C_SS_P_ | 1.91 | 1.85 | 1.68 | 1.75 | 1.42 | 1.47 | |
|  | C_dummySS_p_/DPQdam | 1.58 | 1.49 | 1.67 | 1.73 | 1.31 | 1.27 | |
|  | C_dummySS_p_/Q | 2.58 | 2.54 | 1.91 | 2.01 | 1.91 | 2.05 | |
|  | C_PS_P_ | 2.46 | 2.39 | 2.11 | 2.18 | 1.80 | 1.79 | |
| PS | C_SS_P_ | 1.61 | 1.41 | 1.46 | 1.49 | 1.24 | 1.16 | |
|  | C_dummySS_p_/DPQdam | 1.54 | 1.28 | 1.51 | 1.56 | 1.24 | 1.13 | |
|  | C_dummySS_p_/Q | 2.46 | 2.23 | 1.72 | 1.73 | 1.78 | 1.77 | |
|  | C_PS_P_ | 2.41 | 2.08 | 1.92 | 1.96 | 1.73 | 1.54 | |
| **Nucleus size: 36 Breeding Queens** | | | | | | | |  |
| SS | C_SS_P_ | 1.55 | 1.67 | 1.36 | 1.34 | 1.16 | 1.27 | |
|  | C_dummySS_p_/DPQdam | 1.27 | 1.33 | 1.34 | 1.34 | 1.05 | 1.09 | |
|  | C_dummySS_p_/Q | 2.09 | 2.21 | 1.54 | 1.54 | 1.55 | 1.68 | |
|  | C_PS_P_ | 1.97 | 2.12 | 1.70 | 1.72 | 1.44 | 1.52 | |
| PS | C_SS_P_ | 1.30 | 1.21 | 1.17 | 1.22 | 0.99 | 0.93 | |
|  | C_dummySS_p_/DPQdam | 1.23 | 1.13 | 1.20 | 1.25 | 0.98 | 0.93 | |
|  | C_dummySS_p_/Q | 1.97 | 1.82 | 1.38 | 1.40 | 1.42 | 1.38 | |
|  | C_PS_P_ | 1.93 | 1.80 | 1.53 | 1.59 | 1.37 | 1.30 | |

Predicted SE: mean prediction (by the inverse averaged information matrix) of the SE of genetic (co)variance estimates over repetitions. Realized SE: SD over repetitions of the error on the variance estimates of worker (${\hat{\sigma}^{2}}_{W}$) and queen (${\hat{\sigma}^{2}}_{Q}$) effects, as well as the covariance ($\hat{\sigma}_{\mathrm{WQ}}$).
Controlled mating strategy in the simulation: SS (single sire) and PS (pseudo sire) mating.
Sire pedigree modeling for controlled mating: C _SS_P_, C_dummySS_P_/DPQdam, C_dummySS_P_/Q , C_PS_P_: controlled mated queens are assigned respectively in the pedigree single sires, dummy single sires per dam of DPQ(s), dummy single sires per mated queen, pseudo sires.
