# Supplementary figures and images for: Uncertainty in the mating strategy of honeybees causes bias and unreliability in the estimates of genetic parameters
Source: Genet Sel Evol. 2024 Apr 17;56:30. doi: 10.1186/s12711-024-00898-3 (PMC11022492; doi:10.1186/s12711-024-00898-3)

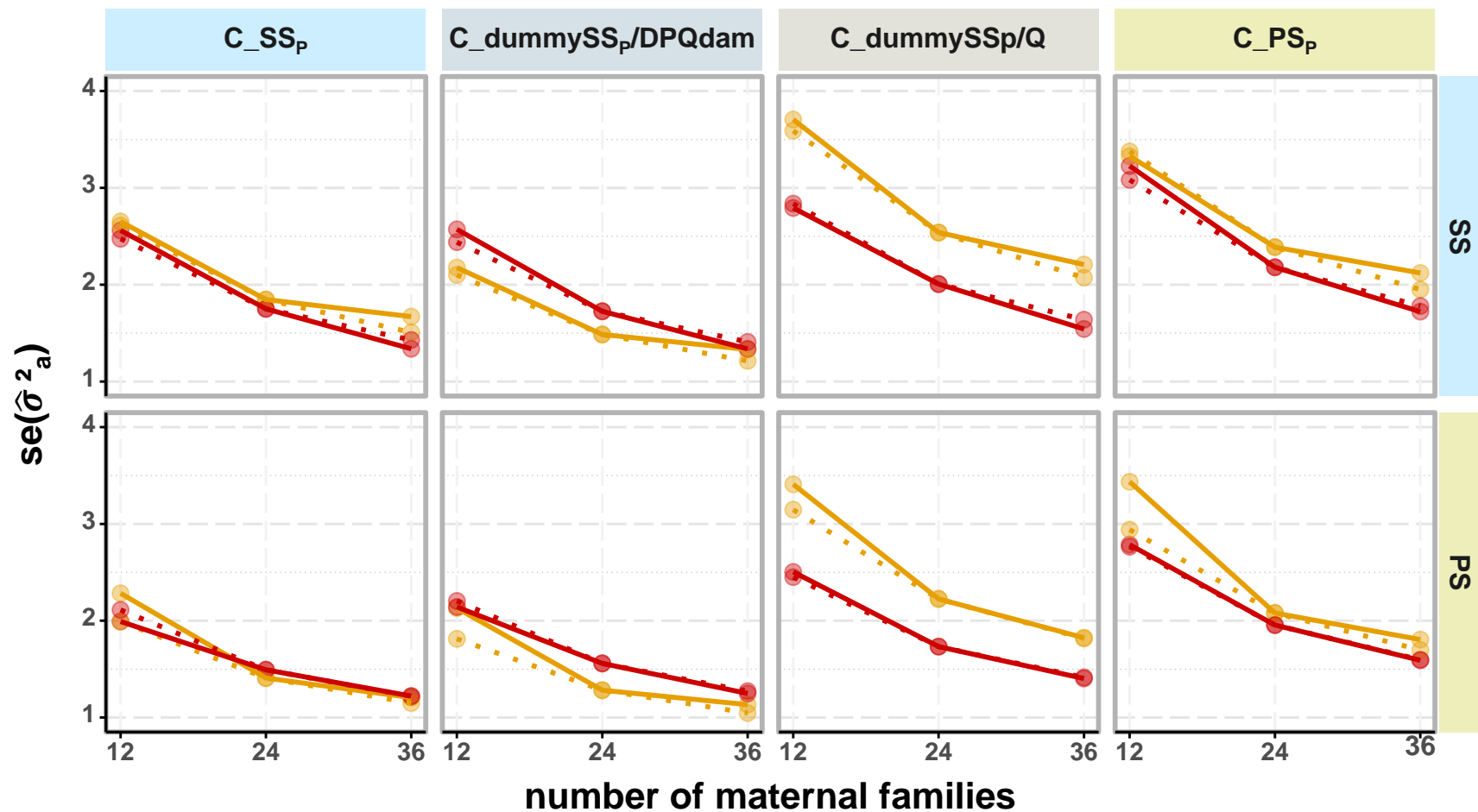

Supplement: Supplementary file 4 — Additional file 4: Figure S1. Realized and predicted (from 24 family scenario results) standard errors of the genetic variance estimates. The first row of graphs is from scenarios using single sire mating (SS) as the controlled mating strategy in the simulation, and the second row from scenarios using pseudo sire mating (PS). From left to right, results were obtained from sire pedigree modeling scenarios for controlled mating assigning single sires (C_SSP); a dummy single sire per dam of DPQs (C_dummySSP/DPQdam); a dummy single sire for each mating (C_dummySSP/Q); and pseudo sires (C_PSP). se (${\upsigma }_{{\text{a}}}^{2}$σa2) is the standard error of estimated genetic variance, for either worker or queen effects (see color legend). Dashed lines link the standard error predicted for N = 12 and N = 36 maternal families, using the values obtained with 24 maternal families, and dividing it by $\sqrt{\frac{{\text{N}}}{2}}$N2. Continuous lines link standard errors obtained when running simulations with all three breeding nucleus sizes: 12, 24 and 36 maternal families. Simulations used genetic parameter set one, with a null genetic correlation between worker and queen effects and equal variances for both effects, using 200 replicates. The predicted and realized standard errors are similar. [file 12711_2024_898_MOESM4_ESM.pdf]
